# Bamboozle: A Bioinformatic Tool for Identification and Quantification of Intraspecific Barcodes

**DOI:** 10.1111/1755-0998.14067

**Published:** 2025-02-04

**Authors:** Matthew I. M. Pinder, Björn Andersson, Hannah Blossom, Marie Svensson, Karin Rengefors, Mats Töpel

**Affiliations:** ^1^ Department of Marine Sciences University of Gothenburg Göteborg Sweden; ^2^ NIRAS Sweden AB Göteborg Sweden; ^3^ Department of Biology Lund University Lund Sweden; ^4^ Bigelow Laboratory for Ocean Sciences Boothbay Maine USA; ^5^ IVL Swedish Environmental Research Institute Göteborg Sweden

**Keywords:** metabarcoding, microalgae, microbial evolution, polymorphism, population genomics

## Abstract

Evolutionary changes in populations of microbes, such as microalgae, cannot be traced using conventional metabarcoding loci as they lack intraspecific resolution. Consequently, selection and competition processes among strains of the same species cannot be resolved without elaborate isolation, culturing, and genotyping efforts. Bamboozle, a new bioinformatic tool introduced here, scans the entire genome of a species and identifies allele‐rich barcodes that enable direct identification of different genetic strains from a population using amplicon sequencing of a single DNA sample. We demonstrate its usefulness by identifying hypervariable barcoding loci (< 500 bp) from genomic data in two microalgal species, the diploid diatom *Skeletonema marinoi* and the haploid chlorophyte *Chlamydomonas reinhardtii* . Across the two genomes, four and twenty‐two loci, respectively, were identified that could *in silico* resolve all analysed genotypes. All of the identified loci are within protein‐coding genes with various metabolic functions. Single nucleotide polymorphisms (SNPs) provided the most reliable genetic markers, and among 54 strains of *S. marinoi,* three 500 bp loci contained, on average, 46 SNPs, 103 strain‐specific alleles, and displayed 100% heterozygosity. This high level of heterozygosity was identified as a novel opportunity to improve strain quantification and detect false positive artefacts during denoising of amplicon sequences. Finally, we illustrate how metabarcoding of a single genetic locus can be used to track abundances of *S. marinoi* strains in an artificial selection experiment. As future genomic datasets become available and DNA sequencing technologies develop, Bamboozle has flexible user settings enabling optimal barcodes to be designed for other species and applications.

## 1 Introduction

Microalgae are a diverse paraphyletic group of aquatic microbes responsible for half of global primary production (Falkowski, Barber, and Smetacek 1998; Field et al. 1998). Many species have enormous population sizes compared with multicellular organisms and display high levels of intraspecific genetic diversity (Flowers et al. 2015; Gollnisch, Ahrén, and Rengefors 2024), although there are also a few notable cases with moderate diversity (e.g., Filatov 2019). Despite their ecological importance, knowledge about individual species' functional diversity and evolutionary potential is limited (Godhe and Rynearson 2017). Microalgal blooms can contain thousands to millions of different clones (Sassenhagen et al. 2021), but designs of evolution experiments are currently constrained to only one or a few strains as they generally utilise clonal cultures (e.g., Lohbeck, Riebesell, and Reusch 2012; Schaum et al. 2017, 2018; Sefbom et al. 2015; Wolf et al. 2019). If these strains are co‐cultured in competition, as is often needed in evolutionary and ecological experiments, the individual strains cannot readily be identified or quantified using existing microscopic or genotyping methods. Currently, genotyping of microalgae relies heavily on microsatellite analysis (Rengefors et al. 2017), although newer sequencing methods such as RAD‐seq provide a method for obtaining fine‐scale genotyping and genomic information (Rengefors et al. 2021). Although suitable for population genetic and genomic studies, these methods still rely on individual DNA samples from each strain for genotype identification. This severely restricts the usefulness of these methods in quantitative evolutionary studies, where strain isolation is time‐consuming or even impossible if the species is difficult to culture (Rengefors et al. 2021) or if the population's clonal diversity is large (Schaum et al. 2017; Scheinin et al. 2015).

An alternative approach to tracking relative cellular abundances directly from a community sample involves using massively parallel amplicon sequencing of a diverse locus, a method known as metabarcoding. The main advantage of such an approach is that it allows the estimation of the relative abundance of millions of individual DNA molecules, and by extension the cells they originate from, from a single DNA sample. This method has revolutionised the field of microbial ecology in the past decade (Hebert et al. 2003; Taberlet et al. 2012). Depending on the taxonomic group and the resolution required, various highly conserved genes are commonly analysed, including ribosomal RNA genes (16S, 18S, 23S, or 28S), mitochondrial cytochrome c‐oxidase subunit 1 (COI), and the chloroplastic ribulose‐1,5‐bisphosphate carboxylase/oxygenase large subunit (*rbcL*) (Guo et al. 2015; Pujari et al. 2019; Yamada et al. 2017). Metabarcoding enables a relatively simple and standardised estimation of community‐wide parameters, such as inter‐ and intra‐specific diversity and the presence of low‐abundance or cryptic taxa. However, while the within‐species conservation of these loci makes them ideal for addressing interspecific questions, this same property restricts their usefulness as intraspecific markers (Canesi and Rynearson 2016; Godhe et al. 2006; Guo et al. 2015; Table S1).

With a sufficiently diverse intraspecific barcode marker, it should be possible to track the abundance and quantify the fitness of individual strains from a mixed microbial population. Such an approach would be analogous to ‘barcoding analysis by sequencing’, or Bar‐seq experiments, where knock‐out mutant lines are generated by inserting constructs with unique synthetic barcodes in the genome (Robinson et al. 2014). Access to naturally occurring intraspecific DNA barcode loci in microalgae and other microbes would provide a powerful tool to address fundamental questions about their evolution and population structures, analogous to how metabarcoding and Bar‐seq have radically changed our understanding of microbial biodiversity and gene function in the last decade.

Genome sequencing projects are enabling us to resolve the genetic diversity between species of microalgae (Armbrust et al. 2004; Bowler et al. 2008; Merchant et al. 2007; Mock et al. 2017; Read et al. 2013; Worden et al. 2009), and population genomic datasets are becoming increasingly available (Blanc‐Mathieu et al. 2017; Flowers et al. 2015; Osuna‐Cruz et al. 2020; Rastogi et al. 2020; von Dassow et al. 2015), providing information about intraspecific genetic diversity. From this data, it is possible to identify novel barcoding loci with intraspecific resolution.

Here we introduce Bamboozle, a bioinformatic approach that uses whole genome sequence data to identify intraspecific barcodes by scanning the entire genome of a species for suitable loci. As a proof of concept, we have identified and designed primers for four barcodes in the diploid marine diatom *Skeletonema marinoi* and four in the haploid model chlorophyte *Chlamydomonas reinhardtii* . Furthermore, we demonstrate how one single barcode locus can be used to track strain selection in an artificial evolution experiment incorporating over fifty strains of *S. marinoi* from two Baltic Sea inlets on the Swedish east coast (Andersson et al. 2024). Based on the data from this experiment, we have identified opportunities, as well as challenges, in employing amplicon sequencing to address questions regarding evolutionary processes in microbes. Although we only demonstrate Bamboozle's applications in microalgae, we envision this method being used to identify intraspecific barcoding loci in any haploid or diploid species if a reference genome and whole genome sequence data from multiple individuals are available.

## 2 Materials and Methods

### 2.1 Bamboozle Workflow

The objective of the Bamboozle tool is to identify novel intraspecific barcoding loci that can distinguish between individual genotypes originating from a common population. To achieve this, the identified loci need to fulfil three criteria as outlined by Kress and Erickson (2008): (1) they should be short enough to allow amplification and sequencing with current technologies, (2) they should have significant interspecific (in our case intraspecific) variability, and (3) they should have conserved flanking regions that allow the binding of primers. Bamboozle takes a similar approach to existing methods for identifying novel barcoding loci by scanning the entire genome (Angers‐Loustau et al. 2016; Paracchini et al. 2017). In contrast to the aforementioned studies, we focus on the identification of barcodes with intraspecific resolution, looking for loci where at least one allele in each strain is not shared by any other strain in the study, thereby allowing us to quantify the relative abundance of each strain. Such unshared alleles are hereafter referred to as ‘strain‐specific alleles’. To that end we applied Bamboozle to 54 recently isolated strains of *S. marinoi* that have been used in a copper tolerance selection experiment (Table S2). For detailed information on the strains and culture conditions, see Andersson et al. (2022, 2024).

**FIGURE 1:**
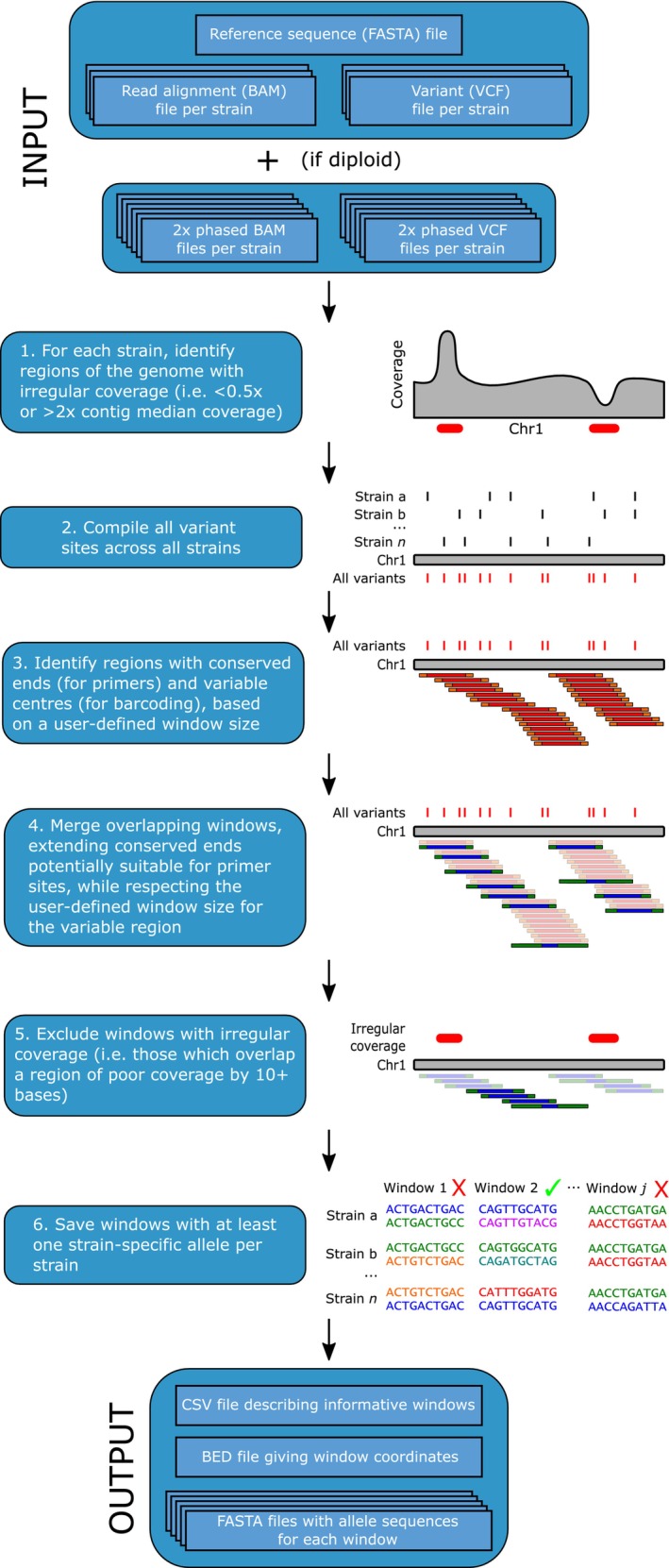
Schematic summarising the steps of the Bamboozle pipeline, as well as input and output files. Each step is described in more detail in the 2. Materials and Methods section.

The steps of the Bamboozle approach for barcode identification are outlined in Figure 1 and described in more detail below. The required input data includes a reference genome for the organism of interest and whole genome sequence (WGS) data from the strains to be analysed. Throughout the paper we use the strain concept (Lakeman, von Dassow, and Cattolico 2009), which for most practical purposes is synonymous with genotype in multicellular organisms. The WGS data from each strain is required in two formats: (1) a sorted BAM file generated by aligning the WGS data to the reference sequence and (2) single nucleotide polymorphism (SNP) data in VCF format generated from the aforementioned BAM file using GATK (Van der Auwera and O'Connor 2020). In the case of diploid species, phased BAM and VCF files (i.e., where data from each allele is stored in separate files) are also required to analyse individual alleles. Bamboozle is developed and tested in Python version 3.7.10 and consists of six main steps, detailed below.

1. *Identification of regions of unusual coverage*. When mapping WGS data to a reference genome, one would expect a similar read coverage across the entirety of the reference. However, while short regions deviating from this expectation could result from potentially informative deletions or insertions in the mapped strain versus the reference, longer such regions could be due to misassembly of the reference genome, repeat regions with significant length differences between strains, or gene duplications. As these regions will artificially inflate the number of SNPs identified in the data, and thereby propose unsuitable barcoding loci (see e.g., barcode *Sm_C2W24* in Data S1), they are masked following analysis with samtools depth (Danecek et al. 2021) and bedtools genomecov (Quinlan and Hall 2010). Regions with a read coverage less than 50% or more than 200% of the contig median for that strain are thereby excluded from the analysis.
2. *Compilation of SNP locations*. Bamboozle parses the VCF files from each strain, extracting the locations of all SNPs.
3. *Genome screening based on conserved sites*. One of the core requirements for a suitable barcode is having conserved flanking regions for primer binding. Bamboozle uses a sliding window approach and traverses each reference sequence (step size 1; window size defined by the user), saving for further analysis windows with variable sites that are flanked by potential primer sites (i.e., regions without any SNPs). The size of the sliding window and the primer sites can be defined by the user with the ‐‐window_size and ‐‐primer_size options, respectively, to account for different amplification and sequencing strategies.
4. *Merging of overlapping windows*. Variable windows are merged if they overlap and are flanked by potential primer sites, reducing the number of windows to screen in the next step (see step 4 of Figure 1).
5. *Exclusion of windows with unusual read coverage*. The merged windows are now compared to the coverage data generated in step 1, and windows that overlap a region of read coverage fluctuation by ten or more bases are excluded. As indel length variations can be potentially valuable in terms of differentiating between strains, allowing short (< 10 bp) stretches of irregular coverage (indicative of short indel regions) is a compromise between the potential for informative loci and the complications such regions may introduce, as noted in step 1.
6. *Identifying informative loci*. samtools faidx and bcftools consensus (Danecek et al. 2021) are applied to each remaining locus to generate consensus sequences for each strain, or each allele in the case of a diploid organism. The individual allele sequences are then analysed to determine if each strain in the dataset contains at least one strain‐specific allele, in which case the locus is reported to the user as a potentially suitable barcoding marker.

As output, Bamboozle reports the proposed barcoding loci in (1) a tab‐separated file, giving coordinates and metadata for each locus, (2) a BED file of locus coordinates, intended for easy visualisation, such as in a genome browser, and (3) a multi‐FASTA file for each locus, containing the sequence of each allele in each strain. This enables quick and easy manual evaluation of the output and design of PCR primers. To aid prospective users in running the Bamboozle pipeline, we have also provided a guide document (Data S2).

### 2.2 Strain Retrieval and Whole Genome Sequencing

Individual strains of *S. marinoi* were germinated from resting stages isolated from sediment using standard micropipetting techniques (Härnström et al. 2011). Surface sediment was collected in June 2017 from two semi‐enclosed inlets of the brackish Baltic Sea, where one (Gåsfjärden [VG]; 57°34.35′N, 16°34.98′E) has been exposed to historical copper mining (Söderhielm and Sundblad 1996) while the other (Gropviken [GP]; 58°19.92′N, 16°42.35′E) has not. A total of 69 and 55 strains were isolated from VG and GP, with 88% and 94% survival, respectively. Strains were cultured in locally sourced seawater (salinity 7), which had been sterile filtered (Sarstedt's [Helsingborg, Sweden] 0.2 mm polyethersulfone membrane filter) and amended with f/2 nutrients (Guillard 1975) and 106 μM SiO_2_. Cultures were continuously screened for contamination by other microalgae and auxospore formation or bimodal cell sizes, which indicate sexual inbreeding in *S. marinoi* (Ferrante et al. 2019), and such cultures were discarded.

DNA extraction was performed within one month of revival from resting stages in a total of 28 strains from GP and 30 from VG (Table S2). Cultures were subjected to a combination of mechanical cleaning (triple washing in 20 μg mL^−1^ Triton X‐100 media and cell collection on a 3.0‐μm polycarbonate filter), followed by a five‐day antibiotic cocktail treatment (90 μg mL^−1^ Paromomycin, 10 μg mL^−1^ Ciprofloxacin, and 40 μg mL^−1^ Cefotaxime). On day six, cells were collected via centrifugation in 50 mL Falcon tubes (10,000 × *g*, for 10 min), flash‐frozen in liquid nitrogen, and stored at −80°C. DNA was extracted using a CTAB‐phenol–chloroform protocol as described in Godhe et al. (2001), but with additional RNA digestion during cell lysis (65°C for 60 min. using 1 mg RNaseA mL^−1^ CTAB buffer). DNA yield was quantified using Qubit (Thermo Fisher Scientific). In two sequential attempts, strains VG1‐2_65 and VG1‐2_99 did not survive the antibiotic treatment, and extractions yielded insufficient amounts of DNA for PCR.

Whole genome sequencing was performed on the remaining 56 *S. marinoi* strains using ½ lane of an Illumina NovaSeq S4 flow cell. Library preparation was done using the PCR‐based Nextera protocol with enzymatic fragmentation. This resulted in 722.82 Mreads (2 × 150 bp paired‐end).

Two of the 56 strains were excluded from the subsequent Bamboozle analysis due to very low WGS read coverage (GP2‐4_44) and being a clone of another strain in the study (GP2‐4_45). The remaining 54 strains were then used for the barcode analysis.

### 2.3 Running the Bamboozle Pipeline on *S. marinoi*

The first 15 bases of the 5′ end were trimmed from the resulting WGS data, based on the results of the FastQC v0.11.9 analysis (Andrews 2010). The trimmed reads were mapped to the *S. marinoi* strain R05AC reference genome version 1.1.9 (accession no. JATAAI000000000.1) using Bowtie2 version 2.3.4.3 (Langmead and Salzberg 2012), and the resulting SAM files were phased, converted to sorted BAM files, and indexed using SAMtools version 1.9 (Danecek et al. 2021). Variant calling was performed using GATK version 4.1.8.0 (Van der Auwera and O'Connor 2020), following the Best Practices recommendations on the tool's website, and the resulting VCF files were indexed using BCFtools version 1.10.2 (Danecek et al. 2021).

Barcodes for *S. marinoi* were obtained using commit 75467da of the Bamboozle main branch, with a ‐‐window_size parameter of 500 and a ‐‐primer_size parameter of 21. The resulting proposed barcodes were also visualised together with the reference genome using IGV version 2.6.3 (Robinson et al. 2011) to check for anomalies. The multi‐FASTA files produced by the pipeline formed the basis for the barcode database used during the selection experiment.

### 2.4 Running the Bamboozle Pipeline on *C. reinhardtii*

Sequence data from seventeen strains of *C. reinhardtii* was downloaded from either the NCBI Sequence Read Archive (SRA; downloaded using the prefetch and fastq‐dump tools from the SRA toolkit version 2.9.6; Leinonen, Sugawara, and Shumway 2011) or the European Nucleotide Archive (ENA; downloaded using the enaDataGet tool from enaBrowserTools version 1.6; https://github.com/enasequence/enaBrowserTools) (Table S3; retrieved 22 February 2021). PCR duplicates were removed using filterPCRdupl version 2.3 (https://github.com/linneas/condetri), after which the data was trimmed with Cutadapt version 3.2 (Martin 2011), using a quality threshold of 30 and a post‐trimming length threshold of either 90 (ENA reads) or 45 (SRA reads).

The trimmed reads were mapped to the following sequences from *C. reinhardtii* : the contig‐level assembly of the reference nuclear genome version 5.5 (accession no. ABCN00000000.2); the mitochondrial genome (accession no. NC_001638.1); the plastid genome (accession no. NC_005353.1); and the mating type minus locus (accession no. GU814015.1). Read mapping, variant calling, and VCF indexing were performed as described above for *S. marinoi*.

Barcodes for *C. reinhardtii* were obtained using commit db64632 of the matt_improvement branch, with a ‐‐window_size parameter of 300 and a ‐‐primer_size parameter of 21. The proposed barcodes were then visualised on the reference genome using IGV version 2.6.3 (Robinson et al. 2011) to check for anomalies.

### 2.5 In Silico Annotation and Assessment of Intraspecific Barcodes

Gene models overlapping the proposed barcoding loci were retrieved from version 5.5 of the *C. reinhardtii* reference genome (https://ensembl.gramene.org/Chlamydomonas_reinhardtii) and version 1.1.9 of the *S. marinoi* reference genome. Functional domains were annotated using NCBI's CD‐Search web tool (Marchler‐Bauer and Bryant 2004). When gene models lacked a functional annotation, putative functions were assigned by a homology search using BLASTp against NCBI's non‐redundant protein (nr) database (Altschul et al. 1990) and comparison of functional domain structure.

To ensure our primers were suitable for non‐Baltic *S. marinoi* strains, we downloaded transcriptome data from three strains (FE7, FE60, and skelA) from the Marine Microbial Eukaryote Transcriptome Sequencing Project (Keeling et al. 2014), aligned them to the *S. marinoi* strain R05AC reference genome using HISAT2 version 2.1.0 (Kim et al. 2019), and manually inspected the proposed primer binding sites using IGV version 2.6.3 (Robinson et al. 2011) to identify variants.

In order to evaluate whether the proposed primers would only amplify DNA from *S. marinoi*, a targeted BLASTn search was used to identify orthologous genes in three species within the order *Thalassiosirales* with reference genomes, namely, *Thalassiosira oceanica* (Lommer et al. 2012), *Thalassiosira pseudonana* (Armbrust et al. 2004), and the closely related *Skeletonema subsalsum* (Sarno et al. 2005) using an in‐house draft genome for the latter (unpublished data).

### 2.6 Confirmation of the Bioinformatic Predictions

Illumina amplicon sequencing was used to assess the sequence accuracy of each of the proposed barcoding loci for the diploid *S. marinoi* and Sanger sequencing for the haploid *C. reinhardtii* . Primers were designed for the 21 bp conserved flanking regions (Table S4) and extended with Illumina adapter sequences for *S. marinoi*. The *rbcL*, 16S, and 18S rRNA genes were used as a positive control of amplification, using primers from Guo et al. (2015), Sundberg et al. (2013), and Stoeck et al. (2010), respectively. The barcodes were amplified from 100 ng DNA and a final primer concentration of 0.1 μM using the Phusion High‐Fidelity PCR Kit (Thermo Scientific) with individually optimised annealing temperatures between 62°C and 65°C. A PCR was run for 30 cycles, with 5 s denaturation (98°C), 5 s annealing, 30 s extension (72°C), and a final 5 min extension at 72°C. The evenness of amplification across strains was qualitatively assessed using gel electrophoresis. Strains with poor barcode amplification were re‐run with either increased DNA concentration or up to 35 PCR cycles. Species specificity of the primers was assessed using DNA from two strains of *Skeletonema subsalsum* , as well as a mixture of other common phytoplankton species from the Baltic Sea (see Data S1). The *C. reinhardtii* primers were tested on two other green algae strains: *Chlamydomonas* sp. strain Kgh 20 and *Microglena* sp. strain Kgh 333.

For the 54 *S. marinoi* strains, the proposed barcoding loci were sequenced individually using 2 × 301 bp reads on 1/10 of a lane on the Illumina MiSeq platform with v3 chemistry. Four PCR reactions were run independently for each barcode, and the products were pooled. Further library preparation, including amplicon size selection > 300 bp, Nextera dual‐indexing, and amplicon sequencing, were performed by SciLifeLab (NGI Stockholm), according to the manufacturer's instructions.

Paired‐end reads were quality filtered and trimmed using Cutadapt version 3.2 (Martin 2011) to remove adapter and primer sequences, with a quality threshold of 28 and a minimum read length of 180 bp. The reads were then merged using BBMerge version 38.86 (Bushnell, Rood, and Singer 2017). Chimaera detection was performed per strain using the uchime2_ref function of Usearch version 11.0.667_i86linux32 (Edgar 2016), with the options ‘‐strand plus ‐mode sensitive’, against a database containing all alleles expected to appear in that strain (see section 2.3. Running the Bamboozle Pipeline on *S. marinoi*). The most abundant sequences corresponding to each locus in the database were taken to be the correct sequences, and the database was manually curated if discrepancies were identified (see 3.3. Accuracy of Bioinformatic Predictions and Amplicon Sequencing of Barcodes in *S. marinoi*).

Amplicon sequences from the individual strains that did not match the expected allele sequences were investigated to determine the sources of error. Identification of truncated sequences, sequences containing Ns, off‐targets, and localisation of sequencing errors and SNPs along the amplicons was performed using a pipeline incorporating Bowtie2 version 2.3.4.3 (Langmead and Salzberg 2012), SAMtools version 1.12, and BCFtools version 1.12 (Danecek et al. 2021).

### 2.7 Performance of Intraspecific Metabarcoding in a Selection Experiment

The quantitative performance of one of the barcoding loci proposed by Bamboozle—*Sm_C12W1*—was validated in a selection experiment using the *S. marinoi* strains listed in Table S2. A detailed description of this experimental design and the results is published in Andersson et al. (2024) and is only presented briefly here. Single‐strain cultures were mixed in equal densities based on relative chlorophyll *a* fluorescence (Varioscan Flash Multimode Reader: ThermoScientific) in two pools according to their origin (Gropviken: GP and Gåsfjärden: VG). In tandem, sub‐samples of cultures were fixed individually in 0.2% acidified Lugol's solution to determine the actual cellular abundances of each strain. To this end, three hundred cells of each strain were counted in a Sedgewick Rafter cell counting chamber (Wildlife Supply Company VR) using an inverted microscope (Axiovert 135, Zeiss) at 200x magnification. The dimensions of twenty cells per strain were also measured at 400x magnification according to Hillebrand et al. (1999) and averaged 0.96 μm^2^ μm^−3^ (range 0.61–1.24, *N* = 58).

During the selection experiment, the two populations were maintained in an exponential growth phase via a semi‐continuous dilution scheme as outlined in Andersson et al. (2020, 2024) through dilutions every third day for a total of 42 days. Five 100 mL replicates per population were grown with toxic copper stress of 8.65 μM CuSO_4_, and five replicates were grown without toxic stress. The dilution bottleneck, i.e., the smallest population size in one replicate bottle, was ~10,000 chains of cells. Cultures were harvested at the start of the experiment and after nine and 42 days of selection. DNA was extracted from experimental samples as for the WGS preparation outlined above, but without the antibiotic treatment. Barcodes were PCR amplified from each replicate timepoint. For the single time 0 sample, two technical DNA extraction replicates × two PCR replicates were processed and analysed in parallel (i.e., *N* = 4 technical replicates). The selection experiment samples were indexed and pooled together with the individual genotype, and sequenced on the same MiSeq flow cell using the remaining 9/10 of the read capacity.

Barcode denoising and quantification were performed using a custom script (available at: https://github.com/topel‐research‐group/Bamboozle) making use of DADA2 version 1.16.0 (Callahan et al. 2016), with a database containing all alleles identified across all strains (see section 2.6 Confirmation of the Bioinformatic Predictions). The DADA2 analysis results in assembled and error‐corrected Amplicon Sequence Variants (ASVs). The term ‘ASV’ represents any sequence assembled from the amplicon sequence data (including true allele sequences, chimaeras and other false positives), and the term ‘ASV counts’ represents the number of observations of a sequence in the data. Different settings and combinations of steps were iteratively explored (described in detail in Data S1). We used four criteria to evaluate the quality of the output by measuring: (1) the number of expected ASVs that were not identified (false negatives); (2) the number of unexpected ASVs (false positives); (3) the effectiveness of the merging of read pairs; and (4) if false positive or false negative observations risked affecting the biological interpretation of the artificial evolution experiment.

The relative abundance of each strain was estimated by summing the number of ASV counts from its two alleles. Counting both strain‐specific and shared ASVs improves strain quantification and helps highlight any false positive artefacts, thereby improving the reliability of the analysis results. While assigning ASV counts of strain‐specific alleles is straightforward (i.e., they can only belong to one strain), assigning ASV counts for shared alleles requires dividing the counts among all strains sharing that allele. We achieved this by applying Equation (1) to the data, as described in detail in Andersson et al. (2024).

(1) $$a_{1}=\mathrm{ASV}\;\text{counts of shared allele}\times \frac{a_{2}}{a_{2}+b_{2}+\ldots +n_{2}}$$

where *a*_*1*_ denotes ASV counts from the shared allele assigned to strain *a*, and *a*_2_ denotes ASV counts from the strain‐specific allele of strain *a*. The ASV counts for the strain‐specific alleles of strains *b*‐*n* are denoted by *b*_*2*_ up to *n*_*2*_, where *n* is the number of strains sharing the allele.

The relative abundance of each strain was then calculated by summing the ASV counts for the two alleles of that strain and dividing by the total ASV counts for all alleles across all strains (Equation 2).

(2) $$\text{Relative abundance of strain}\;a=\frac{\;a_{1}+a_{2}}{a_{1}+a_{2}+b_{1}+b_{2}+\ldots +n_{1}+n_{2}}$$

In the two instances where triploids were identified, all alleles were strain‐specific; thus, only two of their respective alleles were included in the calculations, which would not adversely affect the calculation of relative strain abundances.

To validate the approach, we assessed how closely the barcode enumeration of strains matched the microscopic cell counts in single‐strain cultures prior to pooling at time zero (described above; Figure S1).

## 3 Results

### 3.1 Performance of the Bioinformatic Pipeline: Millions of Potential Barcoding Loci Reduced to Twenty‐Six

**FIGURE 2:**
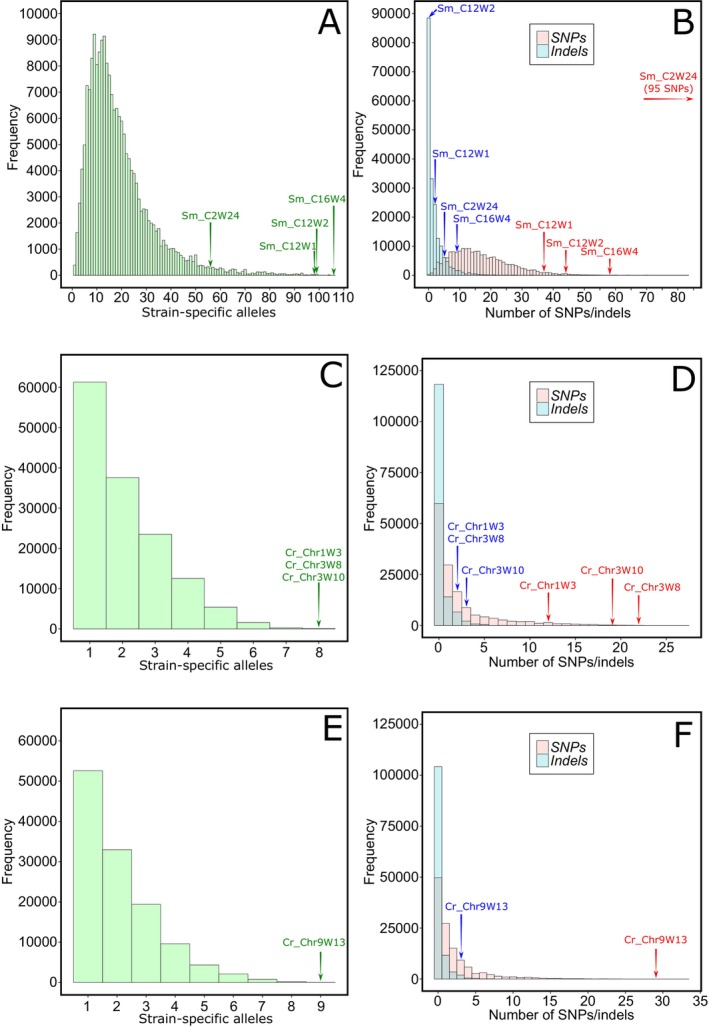
Histograms showing the predicted number of strain‐specific alleles (panels A, C, E) and predicted frequency of SNPs and indels (panels B, D, F) at each window of 500 bp (*S. marinoi*) or 300 bp ( *C. reinhardtii* ). Shown are data from all genomic windows that passed Bamboozle's filters for coverage depth and flanking region conservation, as described in the 3. Results section. In the case of *S. marinoi*, only nuclear contigs are included. Panels A and B depict figures from *S. marinoi*; panels (C, D) depict figures from *C. reinhardtii* mating type +; panels (E, F) depict figures from *C. reinhardtii* mating type‐. Arrows indicate the relative locations of the selected barcoding loci in terms of predicted SNP (red) and indel (blue) frequency and strain‐specific alleles (green). Note that *Sm_C2W24* was predicted with an earlier version of Bamboozle, resulting in deviant values compared to other loci.

We applied Bamboozle to WGS data from 54 strains of *S. marinoi* to identify novel barcoding loci for use with 2 × 301 bp paired‐end sequencing. Initial attempts to identify suitable loci of 300–400 bp failed in this species (data not shown), but Bamboozle was able to propose suitable loci once the window size parameter was increased to 500 bp. Within the *S. marinoi* genome, most (99.5%) of the 54,753,029 possible 500 bp windows were filtered out due to either coverage fluctuations (Table 1) or lack of potential primer sites. Of the remaining 263,000 windows, the average frequency of SNP positions was 12 ± 11, and the observed average number of alleles for the 54 strains was 14 (Table 1). Four potential barcoding loci were proposed that contained at least one strain‐specific allele per strain and passed all filters (Table 1). Three of these, plus an additional hypervariable locus (*Sm_C2W24*) identified prior to the implementation of the coverage filter step (step 1, Figure 1), were selected for further evaluation. Compared to other 500 bp windows passing the read depth and conserved flanking region filters, three barcodes proved especially rich in terms of SNPs and strain‐specific alleles (Figure 2A,B). *Sm_C2W24* was incorrectly predicted to contain an even higher SNP frequency (Figure 2B), due to the presence of a variable length repeat region (see Data S1).

**TABLE 1:** Statistical parameters from Bamboozle's genomic scan for barcode windows. Shown are averages (standard deviations) for the variable barcoding loci identified by Bamboozle (selected windows), with regard to the number of predicted SNPs, indels, and strain‐specific alleles per locus, in contrast to average statistics for the genome as a whole, obtained using a sliding‐window approach (other windows). Where two sets of values are given, the first gives statistics for those windows where only the coverage filter is applied, and the second (in square brackets) gives statistics for those windows where both the coverage filter and the conserved end region filter are applied.

	*S. marinoi*	*C. reinhardtii*	
		Mating type+	Mating type−
Reference genome size (nuclear, mitochondrion, and plastid)	54,794,446 bp	107,613,365 bp	
Number of strains included	54	8	9
Length of genome conforming to coverage filtering	4,740,972	630,835	464,867
SNP density of selected windows	45.0 (9.13)^a^	17.4 (3.25)	22.86 (4.74)
SNP density of other windows	26.26 (14.53) [12.36 (10.83)]	5.30 (6.02) [2.17 (3.42)]	4.68 (5.87) [1.92 (2.95)]
Indel density of selected windows	2.75 (4.27)	1.47 (0.83)	1.71 (1.98)
Indel density of other windows	3.27 (4.55) [1.38 (2.76)]	0.53 (1.03) [0.27 (0.73)]	0.46 (0.98) [0.23 (0.67)]
Number of strain‐specific alleles in selected windows	100 (4.27)	8 (0)^b^	9 (0)^b^
Number of strain‐specific alleles in other windows	30.56 (20.41) [14.30 (13.89)]	2.87 (1.58) [2.08 (1.23)]	2.99 (1.85) [2.11 (1.31)]

^a^ Note that in the case of *S. marinoi*, these statistics exclude *Sm_C2W24* (identified using an older version of the pipeline) but include *Sm_C12W3* (identified in the more recent version).

^b^ When comparing all *C. reinhardtii* selected windows among all 17 strains (i.e., not separated by mating type), there are 12.55 (1.79) alleles per locus.

The Bamboozle tool was also applied to seventeen haploid *C. reinhardtii* strains to identify loci of ~300 bp. While the initial analysis on all strains returned no suitable loci, separation by mating type (eight mt+ strains and nine mt‐ strains) was successful. From a reference genome of 107,613,365 bp (nuclear genome, mitochondrion, plastid, and mt‐ locus [Merchant et al. 2007]), with 107,161,335 potential 300 bp windows, only 137,000 loci (0.13%) contained less than two‐fold coverage deviation compared with the contig median (Table 1) and had conserved flanking regions. About 40% of remaining windows contained only one allele in the population, and less than 1% contained more than seven (Figure 2C,E). Only 22 of these loci were identified by Bamboozle as having one strain‐specific allele for all analysed genomes—fifteen loci for mt+ and seven for mt‐ (Table S5). In addition to this diversity, the proposed loci also contained a much higher number of SNPs than other loci across the genome (Figure 2D,F).

### 3.2 In Silico Annotation of Intraspecific Barcodes and Common Features

All barcodes proposed for both *S. marinoi* and *C. reinhardtii* were inside predicted protein‐coding genes (Table S5). Several barcodes were also in close physical proximity within the same or adjacent genes, e.g., *Sm_C12W1*, *−2*, and *−3*, located within a 2197 bp region containing two gene models (QTG54_008021 and QTG54_008022), as well as *Cr_Chr9W‐12* and *−13* inside gene model *CHLRE_09g389134v5*, and *Cr_Chr11W‐16, −17*, and *−18*, inside gene model *CHLRE_11g467528v5*. The predicted function of the proteins did not have a consistent pattern across the two species and ranged from ribosomal proteins (QTG54_009562 [ribosome biogenesis protein WDR12] and *CHLRE_10g447800v5* [60 kDa SS‐A/Ro ribonucleoprotein]), to enzymes (*CHLRE_03g207250v5* [putative glutamine synthetase] and *CHLRE_13g592050v5* [allantoinase]), and ion transporters (*CHLRE_11g467528v5* [calcium channel]). Eleven barcodes were located inside genes without conserved domains or with conserved Domains of Unknown Function (DUF).

**FIGURE 3:**
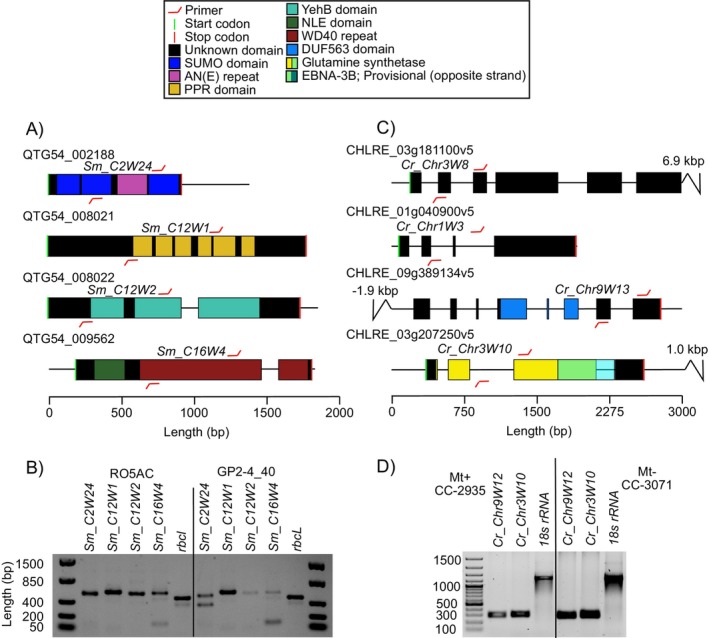
Genomic location of the four barcoding loci identified in *S. marinoi* and selected four loci in *C. reinhardtii*, corresponding to those that primers were designed and evaluated for (for a complete list of all loci and brief gene model annotations, see Table S5). (A, C) Gene models with functional domains, primer sites, and variable regions in *S. marinoi* and *C. reinhardtii*, respectively. (B, D) Gel electrophoresis of PCR products in two strains of *S. marinoi* and one strain each of mating type + and — in *C. reinhardtii*, of loci with good amplification results. AN(E), The three amino acids Alanine, Asparagine, and Glutamic acid [occasionally replaced by Glycine]; DUF563, Domain of Unknown Function; EBNA‐3B, PHA03378 superfamily domain; NLE, NUC135 domain located in N terminal of WD40 repeats; PPR, Pentatricopeptide [RNA binding]; SUMO, Small Ubiquitin‐like Modifier; WD40, Domain involved in various cellular functions including signal transduction, pre‐mRNA processing, and cytoskeleton assembly; YehB, Uncharacterised membrane protein [partial, UPF0754 family] (located on the reverse strand). Glutamine synthetase is represented by its PLN02284 superfamily domain and overlap with EBNA‐3B is shown in green. Note that the primer symbols are not drawn to scale, but the tip of each symbol indicates the exact location where they end and the variable region begins.

Four barcodes from each species were selected for primer design and further barcode development (Figure 3). Among these, no part of the ~500 bp proposed barcoding loci in *S. marinoi* overlapped an intron. Instead, three out of four loci spanned domains with repetitive amino acid regions (*Sm_C2W24*, *Sm_C12W1*, and *Sm_C16W4*), with the conserved flanking regions anchored inside the same or flanking conserved domains (Figure 3A). In contrast, for *C. reinhardtii*, the major part of each ~300 bp proposed locus overlapped an intron, while the conserved flanking regions were located either in introns or exons (Figure 3C). A more detailed annotation of the barcode‐containing genes is provided in Data S1.

As future barcode applications may involve co‐cultivation with other species, we evaluated to what extent the primers were species‐specific. Homologous genes for the proposed loci in *S. marinoi* were identified in three other species of centric diatoms within the order *Thalassiosirales* ( *Thalassiosira pseudonana* , *Thalassiosira oceanica*, and *Skeletonema subsalsum* ) (Table S6). In the two *Thalassiosira* species, each locus's primer sites contained mismatches in at least three positions. However, in *S. subsalsum,* the *Sm_C12W2* primers contained only one mismatch, resulting in a positive PCR amplification. None of the primer pairs amplified in the eleven other Baltic Sea phytoplankton species tested (Figure S2A,B). Although all 54 strains of *S. marinoi* originated from the Baltic Sea, the primer sites were also conserved in three strains from the American Atlantic Coast and the Mediterranean Sea (determined *in silico*; see Table S6), suggesting that the barcodes will function outside the local Baltic Sea population. PCRs of the proposed *C. reinhardtii* loci *Cr_Chr9W12* and *Cr_Chr3W10* did not amplify in two other chlorophytes, including another *Chlamydomonas* sp. strain, suggesting they could also be species‐specific (Figure S2C).

### 3.3 Accuracy of Bioinformatic Predictions and Amplicon Sequencing of Barcodes in *S. marinoi*

The allele sequences predicted by Bamboozle were validated using amplicon sequencing of individual strains of *S. marinoi* or Sanger sequencing for the haploid *C. reinhardtii* . This was done for two reasons: firstly, to generate the accurate allele sequences in the strains and contrast this with Bamboozle's predictions based on genomic shotgun sequences, and secondly, to assess what PCR and sequencing artefacts were prevalent (described in Data S1).

**FIGURE 4:**
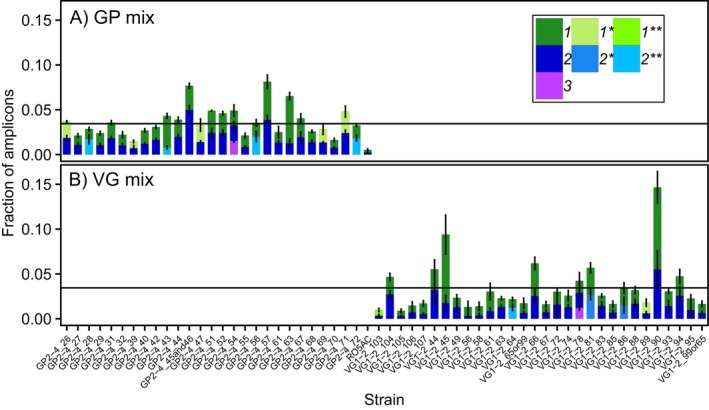
Metabarcoded relative abundance of strains' alleles using the barcode locus *Sm_C12W1*. Shown are two mixed populations of *S. marinoi*. (A) a mixture of 28 strains from Gropviken (GP), and (B) 30 strains from Gåsfjärden (VG). Legend numbers indicate whether this is the first, second, or third allele in each strain, with colours/asterisks indicating if alleles are homologous in two (*) or three (**) other strains. For asterisk‐marked alleles, the abundance has been partitioned between strains using the equations described in Materials and Methods. The horizontal line indicates the expected fraction of amplicons (allele 1 + 2) per strain, assuming even cell densities. Two separate DNA extractions were done on each population, followed by two PCR replicates per extraction. Error bars show standard deviation, with *N* = 3 technical PCR replicates for panel (A), and *N* = 4 for (B). Data has been denoised using the settings for ‘Relaxed DADA2 on amplicons’ as outlined in the Data S1.

All strain‐specific sequences in three of the four sequenced *S. marinoi* loci (excluding *Sm_C2W24* due to abundant indels; see Data S1), as well as *Cr_Chr9W12* and *Cr_Chr3W10* in *C. reinhardtii* , were predicted with > 99.9% accuracy (Table 2). Looking into the reason for the remaining discrepancies, we discovered that some of the strains were triploid (GP2‐4_54 and VG1‐2_78; Figure 4), confounding the software's expectations of diploidy. In other cases, the inaccuracies were caused by insufficient filtering of the variant calling data, resulting in spurious base‐calling errors.

**TABLE 2:** Accuracy of Bamboozle's allele sequence predictions for selected barcodes in *S. marinoi* and *C. reinhardtii* . ‘Predictions’ corresponds to Bamboozle's output sequences based on WGS data, and ‘observations’ are confirmations based on targeted PCR‐based sequencing. *C. reinhardtii* loci with poor amplification and lack of Sanger sequencing data are shown as n/a. *Sm_C2W24* results marked with * indicate results from an earlier iteration of the pipeline, which didn't take coverage or allele phasing into account and was variant‐called using BCFtools (cf. GATK in the most recent iteration).

*Skeletonema marinoi* ^a^	*Sm_C2W24*	*Sm_C12W1*	*Sm_C12W2*	*Sm_C16W4*
Gene model	QTG54_002188	QTG54_008021	QTG54_008022	QTG54_009562
Barcode length in reference	471 (471/471)	523 (523/523)	494 (494/494)	504 (501/504)
(2 × alleles in ref. genome)				
Predicted SNP positions	95*	37	44	58
Predicted indel positions	5*	2	0	9
Predicted strain‐specific alleles	56*	98	99	106
Observed SNP positions	19	38	44	54
Observed indel positions	198	0	0	18
Mean WGS sequencing depth	49 (13–101)	60 (17–140)	59 (17–131)	63 (19–131)
(range, *N* = 54)				
Mean amplicon sequencing depth (range, *N* = 55)	5391 (5–35,434)	4156 (15–10,934)	1693 (5–6960)	3591 (6–19,143)
Accuracy of prediction across all strains and bases	82.65%	99.91%	99.93%	99.88%

*Chlamydomonas reinhardtii* ^b^	mt+			mt−
	*Cr_Chr1W3*	*Cr_Chr3W8*	*Cr_Chr3W10*	*Cr_Chr9W12*
Gene model	CHLRE_01g040900v5	CHLRE_03g181100v5	CHLRE_03g207250v5	CHLRE_09g389134v5
Barcode length	298	297	299	290
Predicted SNP positions	21	23	28	32
Predicted indel positions	4	2	8	3
Observed SNP positions	n/a	n/a	27	32
Observed indel positions	n/a	n/a	8	3
Mean WGS coverage (range *N* = 19)	29 (11–48)	37 (17–58)	32 (16–52)	33 (14–53)
Accuracy of prediction across all strains and bases	n/a	n/a	99.98	99.88

^a^ One strain (GP2‐4_32) was excluded from this analysis due to a failed sequencing.

^b^ One strain (CC‐2938) was excluded from this analysis due to failed DNA extraction.

Most amplicon sequence errors could be either corrected or filtered out using the denoising algorithm of DADA2 (Callahan et al. 2016). We fine‐tuned the settings for the 523 bp *Sm_C12W1* locus to minimise false negative (expected alleles that did not appear as ASVs) and false positive (any ASVs not corresponding to a biological allele) observations (Table S7; section *Attempted optimisation of DADA2 parameters* in Data S1). Ultimately, we identified settings that allowed us to accurately enumerate the relative abundance of strains in a mixed DNA sample using the *Sm_C12W1* locus (Figure 4).

### 3.4 Implementation of an Intraspecific Metabarcoding Method in a Microbial Evolution Experiment

The quantitative performance of *Sm_C12W1* as a metabarcoding locus for *S. marinoi* was further evaluated in an artificial evolution experiment. In the experiment, 30 strains from Gåsfjärden (VG) and 28 strains from Gropviken (GP) were density normalised (relative cell abundance of each strain was 3.4% ± 0.89% based on cell counts using microscopy), mixed in two pools according to their origin, and subjected to 42 days (50–100 generations) of selection with and without toxic copper stress. This data was used to evaluate the quantitative performance of *Sm_C12W1*'s ability to track relative strain abundances.

Despite our expectations of diploidy in the *S. marinoi* strains, there were signs of polyploidy in the experimental data. Some strains exhibited a skewed allele ratio closer to 1:3 (GP2‐4_43, GP2‐4_55, GP2‐4_63, VG1‐2_45, and VG1‐2_61; Figure S3), suggesting that they could be polyploids or that the PCR reaction amplified alleles with slightly different efficiencies. Inspection of WGS read mapping data for these strains showed read coverage at this locus to be consistent with the rest of the genome; thus we consider polyploidy a more likely explanation than gene duplication, which would have been filtered out early in the Bamboozle pipeline (see step 1 of 2.1 Bamboozle Workflow). The combined allele abundance of strains was sometimes over‐represented two‐ to three‐fold (e.g., GP2‐4_57, VG1‐2_45, VG1‐2_90; Figure 4), while others were under‐represented at a similar magnitude (VG1‐2_103, VG1‐2_105, VG1‐2_56; Figure 4). This could not be explained by differences in cell density (*R* ^2^ = 0.0003, slope = −0.0059, *N* = 58) (Figure S1), again suggesting ploidy differences or different DNA extraction efficiencies between cells from different strains. To rule out the latter, we did two technical replicates of the DNA extraction for the start of the selection experiment, which did not result in any markedly different outcomes (Figure 4). Despite these subtle deviations from expectations, the barcode *Sm_C12W1* could be used to identify all strains at close to the expected relative abundances, most often with the two alleles at close to a 1:1 ratio (Figure 4). As the populations evolved under copper stress, one or two strains ultimately became dominant in each treatment (see Figure 4 in Andersson et al. 2024). During this process, the allelic ratio of most strains was observed to be at or near a 1:1 ratio (Figure S3). These observations show that relative changes in ASV frequencies should reflect changes in the relative abundance of strains.

## 4 Discussion

By scanning the entire genome for suitable loci, we illustrate how the Bamboozle approach can identify hyper‐variable barcodes with intraspecific resolution. In the case of both *S. marinoi* and *C. reinhardtii* , most of the genome was not suitable for barcoding and was effectively filtered out by the algorithm. Of the remaining loci fulfilling the criteria for barcodes (Kress and Erickson 2008), only a fraction contained sufficient diversity such that the relative abundance of all strains in the sample could be quantified, highlighting the power of a whole‐genome scan approach for the development of novel intraspecific barcodes. Using a combination of original (54) and published (17: Flowers et al. 2015; Ness et al. 2016) WGS datasets, we illustrate how such intraspecific barcodes can be designed from either publicly available sequence data or through the generation of new data for a species or population of interest.

Within our amplicon sequencing data, many amplicons (~40%) did not correspond to any allele in our database. Therefore, we attempted to identify the sources of these artefacts and how to address them. This was done in order to make recommendations for future studies using workflows similar to ours. First, we note that the 523 bp *Sm_C12W1* locus, paired with Illumina MiSeq's 2 × 300 bp sequencing technology, creates some challenges that may not be encountered in shorter loci. For example, we could not use DADA2 as a stand‐alone tool to process reads since it truncated all amplicons during merging (Table S7). BBMerge performed better but still failed to merge 78% of all reads, due to the short overlapping region and low base‐call quality at the 3′ end of the reads. By pairing BBMerge with DADA2, we found a flexible approach that could correct 99%–99.97% of all sequencing errors in reads that had merged, with only spurious false‐positive allelic observations (Figure S3). Although a shorter locus is preferable and will likely perform better than this, our analysis shows that the tools available to make robust biological inferences from noisy amplicon data extend to 500 bp barcode loci, albeit with a loss of four‐fifths of the sequence data. If future studies cannot identify window sizes smaller than 500 bp, accurate long‐read sequencing of barcodes (e.g., PacBio's Circular Consensus Sequencing technique [Rhoads and Au 2015]) or paired‐end 300 bp sequences from the newer platform Illumina NextSeq could be options worth considering. Long‐read sequencing, in combination with DADA2's error correction, has enabled differentiation between pathogenic and benign *E. coli* strains using single SNP markers and metabarcoding of the whole ~1450 bp 16S rRNA gene (Callahan et al. 2019). Such a sequencing approach opens up the potential for loci of several kb to be used as intraspecific barcodes.

In contrast to sequencing errors, PCR chimaeras provided a more problematic type of artefact. We found that PCR chimaeras could not be removed effectively using software built for processing metabarcoding data, such as Mothur (Rognes et al. 2016; Schloss et al. 2009), UCHIME2 (Edgar 2016), or DADA2's chimaera removal algorithm (Callahan et al. 2016). Exacerbating this issue is the potential for ‘perfect fake’ sequences (chimeras identical to another, non‐chimeric sequence in the dataset [Edgar 2016]). Out of the 110 alleles in locus *Sm_C12W1*, we identified several cases of such ‘perfect fakes' (e.g., allele 1 in GP2‐4_44; Figure S3), and these instances should become progressively more numerous if more strains and alleles are included in the experiments and analysis. However, we could identify these PCR chimaeras in our experimental design primarily by looking at deviation from the expected allelic ratios of individual strains (Figure S3). Depending on the research question and experimental design, chimaeras can be more or less effectively filtered out using similar approaches in future studies. If chimaeras risk causing erroneous biological interpretations from the data, approaches to reduce the number of chimaeras produced include optimising a low PCR cycle threshold (Smyth et al. 2010) or using chimaera‐free PCR kits, such as emulsion PCR (Williams et al. 2006).

The allele phasing step (upstream of Bamboozle) also created some problems, as it could not handle cases of triploidy (Figure 4). Haplotype phasing of polyploids is nontrivial and benefits greatly from long‐read data (Abou Saada et al. 2021). However, as long as polyploidy is accurately identified (e.g., by amplicon sequencing of each strain individually), it should not provide any issues with downstream analysis of strain abundances and may even aid in providing increased allelic diversity for quantification purposes. Consequently, our evaluation of Bamboozle as a tool to identify intraspecific barcodes suggests that it can accurately locate such hypervariable regions. Our quantitative analyses show that with appropriate choice of barcode lengths, PCR kits, sequencing platform, and denoising strategies, amplicon sequencing of intraspecific barcodes can be used to track the relative abundances of large numbers of strains from mixed DNA samples.

Using the model organisms *C. reinhardtii* and *S. marinoi*, we illustrate how the Bamboozle tool is able to identify suitable loci in both haploid and diploid organisms with an available reference genome and whole genome sequencing data from multiple strains. Although further tests are needed to assay the performance of Bamboozle in new populations, as well as the amplicon sequencing performance of the *C. reinhardtii* loci, our results suggest that intraspecific metabarcoding can simplify or enable novel studies of microbial ecology and evolution. These may include determination of genotype diversity in natural populations, studies of ancient DNA from sediment cores (Adams et al. 2019; Härnström et al. 2011), mesocosm incubations of natural phytoplankton communities (Scheinin et al. 2015; Tatters et al. 2013), effects of predation (Sjöqvist et al. 2014), nutrient competition within (Collins 2011) and between species (Descamps‐Julien and Gonzalez 2005), or other drivers that are challenging or impossible to observe in monoculture experiments (Baert et al. 2016; Collins and Schaum 2021).

## Author Contributions

B.A., K.R., M.I.M.P., and M.T. designed the experiments. B.A. designed PCR primers and performed wet lab work related to *S. marinoi*. H.B. and M.S. designed PCR primers and performed wet lab work related to *C. reinhardtii* . M.I.M.P. wrote the barcode identification pipeline. B.A., M.I.M.P., and M.T. analysed the data. B.A. and M.I.M.P. co‐wrote the first draft of the manuscript, and all authors contributed to the final version of the manuscript.

## Conflicts of Interest

The authors declare no conflicts of interest.

## Supporting information


Data S1.

Data S2.

Figure S1.

Figure S2.

Figure S3.

Figure S4.

## Data Availability

The code for Bamboozle is available at https://github.com/topel‐research‐group/Bamboozle, the code for the downstream analyses is available at https://github.com/Bearstar85/Cu_evolution, and the WGS and amplicon sequencing data have been deposited at NCBI under BioProject PRJNA939970. The experimental data has also been uploaded to the Swedish National Data Service at https://doi.org/10.5878/9y59‐7a50 (Andersson et al. 2022).
