# Supplementary material for: Bamboozle: A Bioinformatic Tool for Identification and Quantification of Intraspecific Barcodes
Source: Mol Ecol Resour. 2025 Feb 4;25(4):e14067. doi: 10.1111/1755-0998.14067 (PMC11969633; doi:10.1111/1755-0998.14067)
Supplement: Supplementary file 1 — Data S1. [file MEN-25-e14067-s006.docx]

Bamboozle Manual

Also available at <https://github.com/topel-research-group/Bamboozle/wiki>

# Part 1: Barcode Search

## A note on runtimes

Both the variant calling and the Bamboozle sections of this pipeline can be time-consuming, depending on the number and size of samples.

- Running GATK on one strain (i.e. diploid dataset plus two phased datasets) took around 24 hours total on 20 CPU cores.
- Running Bamboozle on 54 Skeletonema marinoi datasets took around 16.5 hours on 40 CPU cores.

## Preparation

Tools used:

- Samtools version 1.12
- BCFtools version 1.12
- GATK version 4.1.8.0
- Bowtie2 version 2.3.4.3

1.) Prepare the relevant index files for your reference genome file using samtools and GATK

samtools faidx reference.fasta

gatk --java-options "-Xmx2g" CreateSequenceDictionary -R reference.fasta -O reference.dict

2.) Generate sorted BAM files (and associated index files) for all of your sequencing files using the bowtie2 -> samtools pipeline

bowtie2 --no-unal -x reference.fasta -1 seq.R1.fastq -2 seq.R2.fastq -S sample.sam

samtools view -@ $NSLOTS -b -o sample.bam sample.sam

rm sample.sam

samtools sort -@ $NSLOTS -o sample_sorted.bam sample.bam

rm sample.bam

samtools index sample_sorted.bam sample_sorted.bai

2.5.) If investigating a diploid, phase the files using samtools phase

samtools phase -b sample_sorted sample_sorted.bam

samtools index sample_sorted.0.bam sample_sorted.0.bai

samtools index sample_sorted.1.bam sample_sorted.1.bai

3a.) For each of your samples, run the GATK variant calling pipeline for either a haploid...

INFILE1=sample_sorted.bam

INFILE2=sample.ReadGroups

INFILE3=sample.g.vcf.gz

OUTFILE1=sample.vcf.gz

OUTFILE2=sample.filtered.vcf.gz

OUTFILE3=sample_sorted.vcf.gz

gatk --java-options "-Xmx4g" AddOrReplaceReadGroups \

-I $INFILE1 \

-O ${INFILE2}.bam \

-LB foo \

-PL ILLUMINA \

-PU bar \

-SM sample

samtools index ${INFILE2}.bam ${INFILE2}.bai

gatk --java-options "-Xmx4g" HaplotypeCaller \

-R reference.fasta \

-I ${INFILE2}.bam \

-O $INFILE3 \

-ERC GVCF \

-bamout sample.realigned.bam

samtools index sample.realigned.bam sample.realigned.bai

gatk --java-options "-Xmx4g" GenotypeGVCFs \

-R reference.fasta \

-V $INFILE3 \

-O $OUTFILE1

bcftools filter --threads $NSLOTS -i "%QUAL>20" -Oz -o $OUTFILE2 $OUTFILE1

ln -s $OUTFILE2 $OUTFILE3

bcftools index --threads $NSLOTS $OUTFILE3

done

3b.) ... or a diploid

for i in "" .0 .1; do

INFILE1=sample_sorted${i}.bam

INFILE2=sample${i}.ReadGroups

INFILE3=sample${i}.g.vcf.gz

OUTFILE1=sample${i}.vcf.gz

OUTFILE2=sample${i}.filtered.vcf.gz

OUTFILE3=sample_sorted${i}.vcf.gz

gatk --java-options "-Xmx4g" AddOrReplaceReadGroups \

-I $INFILE1 \

-O ${INFILE2}.bam \

-LB foo \

-PL ILLUMINA \

-PU bar \

-SM sample

samtools index ${INFILE2}.bam ${INFILE2}.bai

gatk --java-options "-Xmx4g" HaplotypeCaller \

-R reference.fasta \

-I ${INFILE2}.bam \

-O $INFILE3 \

-ERC GVCF \

-bamout sample.realigned${i}.bam

samtools index sample.realigned${i}.bam sample.realigned${i}.bai

gatk --java-options "-Xmx4g" GenotypeGVCFs \

-R reference.fasta \

-V $INFILE3 \

-O $OUTFILE1

bcftools filter --threads $NSLOTS -i "%QUAL>20" -Oz -o $OUTFILE2 $OUTFILE1

ln -s $OUTFILE2 $OUTFILE3

bcftools index --threads $NSLOTS $OUTFILE3

done

You should now have the following files:

**One per analysis**

| **File** | **Haploid?** | **Diploid?** | **Description** |
| --- | --- | --- | --- |
| reference.fasta | Y | Y | Reference genome sequence in FASTA format |
| reference.fasta.fai | Y | Y | Index file for the reference FASTA |
| reference. dict | Y | Y | Dictionary file for the reference FASTA |

**One per sample being analysed**
Note: All files for a single sample should be in the same (sub)directory; whichever directory sample_sorted.bam is in, Bamboozle will look for all other files related to that sample in that directory.

| **File** | **Haploid?** | **Diploid?** | **Description** |
| --- | --- | --- | --- |
| sample_sorted.bam | Y | Y | Unphased read mapping file (sorted) |
| sample_sorted.bai | Y | Y | Index file for unphased BAM file |
| sample_sorted.vcf.gz | Y | Y | Compressed variant-calling file for unphased BAM |
| sample_sorted.vcf.gz.csi | Y | Y | Index file for variant-calling file for unphased BAM |
| ---------------------------- | ---------- | ---------- | ----------------------------------------------------------- |
| sample_sorted.0.bam | N | Y | First phased read mapping file (sorted) |
| sample_sorted.0.bai | N | Y | Index file for first phased BAM file |
| sample_sorted.0.vcf.gz | N | Y | Compressed variant-calling file for first phased BAM |
| sample_sorted.0.vcf.gz.csi | N | Y | Index file for variant-calling file for first phased BAM |
| ---------------------------- | ---------- | ---------- | ----------------------------------------------------------- |
| sample_sorted.1.bam | N | Y | Second phased read mapping file (sorted) |
| sample_sorted.1.bai | N | Y | Index file for second phased BAM file |
| sample_sorted.1.vcf.gz | N | Y | Compressed variant-calling file for second phased BAM |
| sample_sorted.1.vcf.gz.csi | N | Y | Index file for variant-calling file for second phased BAM |

## Running Bamboozle

Tools used:

- Python 3
- Samtools version 1.12
- BCFtools version 1.12
- Bedtools2 version 2.27.1

Non-standard Python packages used

- [Levenshtein](https://maxbachmann.github.io/Levenshtein/installation.html)

1.) Run the following command:

bamboozle.py barcode -f reference.fasta \

-b sample1_sorted.bam sample2_sorted.bam [...] \

-o Live2Tell_p21_w500_GATK \

--ploidy XXploid

| **Required flags** |  |
| --- | --- |
| -f, --ref | Reference genome in FASTA format |
| -b, --bamfile | Alignment file in BAM format (one per sample, space-separated list) |
| -o, --outprefix | Prefix for output files |
| --ploidy | Ploidy of the target organism (either haploid or diploid) |

| **Optional flags** |  | **Default** |
| --- | --- | --- |
| --window_size | Size of window to scan for variants (inc. terminal conserved regions ['primer sites']) | 500 |
| --primer_size | Size of desired conserved regions at each end of the variable region | 21 |
| -t, --threads | Number of threads for parallelisation | 1 |
| --resume | If a run is interrupted, use this flag to resume | - |

## Output

| **File** | **Description** |
| --- | --- |
| <outprefix>.bed | A BED file containing the coordinates of potential barcode regions |
| <outprefix>.txt | A tab-separated CSV file containing additional information on the potential barcode regions (coordinates, min./max. differences between strains, reference sequences for conserved and variable regions) |
| <outprefix>_alleles/ | A directory containing a FASTA file for each potential barcode region |
| <locus>_alleles.fasta | A FASTA file in <outprefix>_alleles/ containing the sequence(s) for each individual at the given locus |
| barcoding.<date>.log | A log file giving details about loci identified at each step in the analysis |
| time.log | A log file showing the time taken for each step in the analysis |

Once you've sequenced one of these barcodes, proceed to quantification.

# Part 2: Barcode Quantification

## Analysing amplicon sequencing data to quantify Bamboozle-derived barcodes

This part of the tutorial will run through how to quantity your barcodes once you have performed amplicon sequencing; the relevant files are found at <https://github.com/topel-research-group/Bamboozle/> in the scripts/BarcodeQuantification subdirectory (also available as a .zip file if you don't want to clone the entire Bamboozle repository).
The steps below will assume a certain file structure as used in this tutorial, but the commands can be adapted to suit your own needs.
The Quantification.sh shell script will run the entire pipeline from QC to visualisation in one go. However, if you wish to perform each step individually (e.g. to inspect the output or customise settings), we present step-by-step instructions below.

## Sample data

We also provide sample data from our own Skeletonema marinoi experiment, to demonstrate how the pipeline can be used. These files are found in the scripts/BarcodeQuantification/sample_data subdirectory. The readme within BarcodeQuantification explains these files.

## Setup step

1.) Move into the BarcodeQuantification directory.

2.) In order to set up a directory structure for the quality control of your data, firstly make a file called index_file.lst in this directory, whose first column is the names of your samples.

3.) Make a symbolic link to your reference FASTA file, containing the barcodes for each of your samples.

ln -s /path/to/myreference.fasta reference.fasta

4.) Make a directory for your data, and move into it.

mkdir 00_data

cd 00_data

5.) Define the **absolute** path to where all of the raw data is kept.

INDATA="/full/path/to/raw/data"

6.) Make a subdirectory for each sample, and create links to the raw data in the relevant subdirectory. This saves space versus copying the data.

while read i; do

SAMPLE=$(echo $i | cut -f1)

mkdir ${SAMPLE}

FWDREADS=$(find "${INDATA}/${SAMPLE}" -type f -name "*R1*fastq.gz")

REVREADS=$(find "${INDATA}/${SAMPLE}" -type f -name "*R2*fastq.gz")

ln -s $FWDREADS ${SAMPLE}/

ln -s $REVREADS ${SAMPLE}/

done < ../index_file.lst

**Result:** You should now have a subdirectory for each of your samples, each containing two links to .fastq.gz files - forward reads, and reverse reads.

## Trim the input data

Tools used (example):

- [Cutadapt version 3.2](https://cutadapt.readthedocs.io/en/stable/installation.html)
- [FastQC version 0.11.9](https://www.bioinformatics.babraham.ac.uk/projects/fastqc/)
- [MultiQC version 1.9](https://multiqc.info/)

As per good practice, perform quality control on the data, being sure to trim off adapters and primer sequences, and check the quality of the resultant sequences. The specifics will ultimately depend on your own data, but as an example, we used this pipeline:

1.) Define the adapters and primers to trim off (examples below are from our S. marinoi experiment).

ILLUMINA="AGATCGGAAGAG"

FWDPRIMER="AGGYTTCGCCTCCTCAAAC"

REVPRIMER="GGCACGATGCACACGCAAAG"

2.) Trim each pair of read files, and produce a FastQC summary file

- Note: the -a/-A flags refer to 3' adapters, the -g/-G flags refer to 5' primers, and -n 2 tells cutadapt to remove two sequences (i.e. adapter AND primer)
- Parameters for quality (-q) and minimum length (-m) can be adjusted according to your requirements)

while read i; do

FWDREADS=$(ls ${i}/${i}*R1*fastq.gz)

REVREADS=$(ls ${i}/${i}*R2*fastq.gz)

cutadapt -a $ILLUMINA -A $ILLUMINA -g $FWDPRIMER -G $REVPRIMER -n 2 -q 28 -m 180 \

-o ${i}/${i}.R1.trimmed.fastq.gz -p ${i}/${i}.R2.trimmed.fastq.gz $FWDREADS $REVREADS

fastqc -o ${i} ${i}/${i}.R1.trimmed.fastq.gz ${i}/${i}.R2.trimmed.fastq.gz

done < ../index_file.lst

The resultant FastQC files can then be summarised in an interactive plot using MultiQC:

multiqc -ip -o MultiQC_Report .

**Result:** Each sample's subdirectory should now include two .trimmed.fastq.gz files for the forward and reverse reads, respectively, as well as associated .zip and .html files for each trimmed file from FastQC.
You should also have a MultiQC_Report directory containing a multiqc_report.html report file (and a multiqc_data subdirectory).

## Pre-merging reads (optional)

Tools used:

- [BBmerge (part of BBmap version 38.86)](https://jgi.doe.gov/data-and-tools/software-tools/bbtools/bb-tools-user-guide/installation-guide/) (requires Java)

Depending on your barcode and sequencing strategy, read merging can either be performed via BBmerge, or in the subsequent dada2 quantification step (see below).
With a long barcode and a short read pair overlap, we opted for BBmerge, retaining fewer reads while getting fewer false positives, but your mileage may vary.

while read i; do

bbmerge.sh in1=${i}/${i}.R1.trimmed.fastq.gz in2=${i}/${i}.R2.trimmed.fastq.gz \

out=${i}/${i}.merged.fastq.gz outu1=${i}/${i}.unmerged.R1.fastq.gz outu2=${i}/${i}.unmerged.R2.fastq.gz

done < ../index_file.lst

**Result:** Each subdirectory should now contain a .merged.fastq.gz file of merged reads, and two files of unmerged reads - .unmerged.R1.fastq.gz and .unmerged.R2.fastq.gz.

## Setup step

1.) Move back up into the BarcodeQuantification directory for the next step

cd ..

2.) Define whether you already performed read merging using BBmerge, or whether you'll perform it in the next step with dada2.

# Either...

MERGE="bbmerge"

# ... or...

MERGE="dada2"

### Denoising reads

Tools used:

- [dada2 version 1.26.0](https://benjjneb.github.io/dada2/dada-installation.html)

The script required for the next step is dependent on whether you ran BBmerge or not.
In either case, the formatting of these scripts borrows heavily from [the nf-core/ampliseq pipeline](https://nf-co.re/ampliseq).

**Important:** Your reference fasta file (which the script expects to be called reference.fasta, though this can be adjusted) should have headers formatted as >ID Genus species, [as described under Formatting Custom Databases in the dada2 manual](http://benjjneb.github.io/dada2/training.html). In our case, as we're working with intraspecific barcodes, we replace the species with strain_allele (e.g. >strain1_1 MySpecies strain1_1).

The script currently assumes that you used the naming conventions implemented above, but this can be changed within the R scripts.

if [ $MERGE == "dada2" ]

then

Rscript rundada2.PE.R

elif [ $MERGE == "bbmerge" ]

then

Rscript rundada2.BBmerged.R

fi

**Result of dada2 analysis:** Regardless of which of the scripts you use, you should have an output directory (name defined by you in the script) containing the following:

|  |  |
| --- | --- |
| 1.stats.tsv | A breakdown of how many read pairs made it through each step in the pipeline |
| ASV_seqs.fasta | Sequences of all the ASVs found across the samples (with md5 hashes as IDs) |
| ASV_table.tsv | Quantities of each ASV found in each sample |
| DADA2_stats.tsv | Same as *1.stats.tsv* |
| DADA2_table.tsv | As ASV_table.tsv, but contains an additional column for the ASV sequence |
| filtered/ | Directory containing sequences filtered by dada2's filterAndTrim, and accompanying summary stats |
| logs/ | Directory containing logfiles and .rds files for each step in the pipeline, to resume analyses in case of failures |
| **strain_table.tsv** | **Quantities of each ASV found in each sample, including exact matches to the reference database** |

## Visualisation

We can now visualise the results contained within strain_table.tsv using the Barcode_analysis.R R script.

**Note:** If you're running this analysis using the sample data, ensure that Allele_indexing.txt and Indexing.txt have been copied into the BarcodingQuantification directory before continuing.

**Important:** This script is currently set up for use with the S. marinoi C12W1 barcode, as described in the publication associated with this repository. However, it can be used as a template for analyses of other species and barcodes.

1.) Make a new directory for the results of Barcode_analysis.R.

mkdir 02_plots

cd 02_plots

2.) Run the analysis script.

Rscript ../Barcode_analysis.R
