# Supplementary material for: Bamboozle: A Bioinformatic Tool for Identification and Quantification of Intraspecific Barcodes
Source: Mol Ecol Resour. 2025 Feb 4;25(4):e14067. doi: 10.1111/1755-0998.14067 (PMC11969633; doi:10.1111/1755-0998.14067)
Supplement: Supplementary file 2 — Data S2. [file MEN-25-e14067-s004.docx]

Supplemental Information

Bamboozle: A bioinformatic tool for identification and quantification of intraspecific barcodes

Matthew I M Pinder, Björn Andersson, Hannah Blossom, Marie Svensson, Karin Rengefors, and Mats Töpel

## Barcode Sm_C2W24

During development of the Bamboozle pipeline, we initially did not include a filter for deviations from expected genomic coverage (steps 1 and 5 in *Bamboozle workflow* [Materials and Methods]). When running this iteration of the pipeline (commit ae28b1b of the Bamboozle main branch; --window_size 500, --primer_size 21) on the *S. marinoi* WGS data, 245 barcoding loci were proposed. For one such barcode – *Sm_C2W24* – Bamboozle predicted 95 SNPs and five indels within our studied population, however manual inspection indicated that some strains had low sequencing coverage across the most variable region. The variant calling algorithm of GATK, and by extension Bamboozle, had misinterpreted a variable-length repeat region for a high abundance of SNPs (Table 2). We opted to sequence this locus to determine whether such a region could indeed serve as a suitable barcoding locus, or whether the presence of micro- or minisatellite-like regions was problematic.

Sequencing of *Sm_C2W24* (as described under *Confirmation of the bioinformatic predictions* in Materials and Methods) revealed that almost all allele sequences in the different strains were incorrectly predicted, and per-base accuracy across the locus was only 82.6%. The alleles of *Sm_C2W24* among the strains in our study showed length differences of 198 bp, caused by variable copy number (2 to 24 copies) of a 9 bp repeat encoding Alanine- Asparagine- Glutamic acid (AN(E)) (length difference also confirmed by gel electrophoresis, Fig. 3B). The presence of this long repeat region greatly complicated merging of the paired-end sequencing reads, to the extent that we could not confirm the true sequences in many strains. Thus, adjustments were made to Bamboozle to filter out similar regions, and *Sm_C2W24,* together with 90-99% of the genomes (Table 1), was disregarded in subsequent analyses.

## Detailed in silico annotation of intra-specific barcode features

All four barcode-containing genes in *S. marinoi* (Fig. 3A) encoded proteins predicted to interact physically with other biomolecules. *Sm_C2W24* was inside QTG54_002188, a gene with a unique domain structure for which we could find no homologs in the NCBI database (analysis performed December 12th, 2021). It contains three repeats of a Small Ubiquitin-like Modifier (SUMO) domain, involved in post-translational protein modification, with the primer sequences anchored in the second and third repeats. SUMO proteins are well characterised in humans (Verger et al. 2003), yeast (Hofmann et al. 2000), and *Arabidopsis* (Morrell and Sadanandom 2019). In these species, the SUMO domain is only presented in a single copy in a short gene, and the ca. 9-11 kDa peptide is covalently attached to primarily lysine residues of proteins to modify their function. Like ubiquitin, the SUMO modification can serve a multitude of different functions, including targeting proteins for degradation, or changing the activity of transcription factors. The centre of the barcode contains a 9 bp repeat encoding Alanine- Asparagine- Glutamic acid [occasionally replaced by Glycine] which we refer to as the AN(E) repeat. The AN(E) repeat of QTG54_002188 has no homologs amongst phytoplankton in NCBI’s current non-redundant protein (nr) database, but it has similarity to viral proteins in *Plasmodium* spp.*,* *Vibrio casei,* and *Cotonvirus japonicus*, which all have genes with an 18mer repeat structure yielding peptides with similar residues (e.g. TNEANE, NETNEV, and ANDAGE).

*Sm_C12W1* was located inside a pentatricopeptide (RNA binding) repeat region of QTG54_008021. The closest BLASTp match in the NCBI database was a ‘maturation of *rbcL* mRNA’ protein in *A. thaliana* (AT4G34830.1). However, these genes are likely not orthologous, as the *S. marinoi* genome has more than 20 genes similar in sequence to QTG54_008021 and AT4G34830.1, and out of these, QTG54_010906 has higher amino acid similarity to AT4G34830.1. Consequently, QTG54_008021 may be involved in mRNA maturation or other types of RNA interactions, but perhaps not specifically for *rbcL*.

*Sm_C12W2* was located inside one of two YheB membrane domains of QTG54_008022*.* This gene is relatively conserved in *T. pseudonana* (Thaps3_5794; 72% coverage and 69% identity at the nucleotide level), but has three and four mismatches in the forward and reverse primer sites, respectively (Table S6). We also found a protein homologous to QTG54_008022 in *T. oceanica* (THAOCEA1_01345), but nucleotide similarity was eroded to the point that we could not accurately identify the primer sites (Table S6).

*Sm_C16W4* was located inside a WD40 repeat of QTG54_009562, whose protein is similar to microtubule-associated ribosome biogenesis protein YTM1 in *S. cerevisiae*. Unfortunately, the *Sm_C16W4* primer set (Table S4) did not amplify well once adapters had been added to primers (Fig. 3B), likely due to increased dimerisation or a modified secondary structure.

## Identification of sources of amplicon errors of barcodes in S. marinoi

During amplicon sequencing of the proposed *S. marinoi* barcoding loci, we encountered a number of difficulties in processing the sequencing data, which we report below to advise users when handling their own data.

Firstly, despite quality filtering (3′ Illumina adapter removal, 3′ quality filter of Phred score 28, minimum post-trim read length 200), sequencing errors were present in most of our data (affecting up to 75% of all amplicons). We tried to increase the Phred quality score filter from 28 to 30, but this resulted in 98% of all reads failing to merge, which was unacceptably high.

Secondly, despite checking the primer sequences using BLASTn against the *S. marinoi* reference genome prior to PCR amplification and sequencing, attempts to amplify some of our loci resulted in substantial amounts of off-target amplification. We also noted the formation of primer dimers. In our study, this seemed to particularly affect *Sm_C12W1* and *Sm_C16W4*; the *Sm_C16W4* off-target/primer dimer was short and removed during size selection or adapter filtering, but *Sm_C12W1* escaped these steps and merged into amplicons, accounting for an average 20.97% of merged amplicons (Fig. S4). The terminal regions of the 358 bp off-target sequences in *Sm_C12W1* don’t match the full length of the primer sequences, and BLASTn does not map the primers sequences to these regions. However, the off-target sequences have BLASTn matches on nine different contigs in *S. marinoi*, which themselves have five- to ten-fold higher coverage depth than the genomic average, suggesting they are repetitive elements occurring throughout the genome. This likely explains why the region amplified despite having a relatively poor predicted annealing of the primers. As with sequencing errors, off-targets lower the number of amplicons per sample, but are relatively simple to remove bioinformatically. Consequently, off-targets are unwanted but of little concern to downstream analyses.

The third issue identified with Bamboozle was the appearance of unexpected triploid strains in our experiment. The default expectation of the phasing and variant calling software used for the *S. marinoi* study was diploidy, and this was the ploidy defined in Bamboozle. Furthermore, as the coverage filter employed looks at the coverage across the genome on a per-contig level, it cannot detect duplication of entire contigs/chromosomes corresponding to aneuploidy, or the presence of full polyploidy. Upon subsequent manual inspection of the WGS results, we identified some of our strains as triploid.

The fourth and most serious problem was the presence of PCR chimeras that the bioinformatic chimera filters failed to remove. The chimera removal software we initially employed (the chimera.vsearch command of Mothur version 1.47.0 [Rognes et al. 2016; Schloss et al. 2009]) only identified an average of up to 0.3% of all merged reads in each barcode as being chimeric. However, as chimera removal software often fails to detect chimeras amongst highly similar sequences (>95% similarity), which included almost all of our alleles, we manually examined some of our results and determined that chimeras were indeed being missed by the software. We thus tried a different tool (the uchime2_ref command of Usearch version 11.0.667_i86linux32 [Edgar 2016]), whose 'sensitive' mode produced a result more in line with our manual observations. It should be noted, however, that this mode of the software can produce false positive results. This analysis gave chimera values of between 0.77% and 4.02% of merged reads (Fig. S4), but manual annotation found that chimeras were still present in the filtered data. As chimeras will duplicate during each PCR cycle, they can rapidly generate many exact copies of an artefact amplicon, in contrast to sequencing errors that are, although not completely randomly created, at least more evenly distributed. Chimeras will also only affect SNP positions across the read. At the same time, sequencing errors are expected to affect SNPs and non-informative sites equally, and the chimeric PCR mechanism is indistinguishable from genomic recombination that generates natural diversity within populations. Consequently, chimeras were strongly overrepresented in the generation of false positive observations in some strains (Fig. S3). However, we found that the diploid nature of vegetative *S. marinoi* strains provided opportunities to detect these chimeras, as well as other artefacts (Fig. S3), and they can be masked in various ways when amplicon counts are translated into cell or strain counts.

Throughout the experiment, most strains experienced negative selection. Below around 10 ASV counts per sample, the DADA2 algorithm started filtering out observations, indicating that false negative artefacts were created. However, DADA2 effectively removed all but four false positive observations in the experiment (alleles of the GP population seen in the VG population, and vice versa; Table S7). The remaining four false positives appeared to be chimeric, as did allele GP2-4_44#1, which displayed a skewed allelic ratio representing up to 5% (>1,000 copies) of amplicons in several replicate samples, while GP2-4_44#2 was not detected at all (Fig. S3). As the first 316 bp of this allele are identical with GP2-4_27#1, and the last 354 bp with GP2-4_27#2 (the two alleles of the most dominant strain in the same samples), there is a central 147 bp region where a PCR chimera from these two alleles would produce GP2-4_44#1. In addition to enabling chimera detection in situations like the one above, heterozygosity (i.e. the presence of two different alleles at a given locus) provided an additional analytical advantage, enabling the use of equations 1 and 2 to accurately partition alleles that were shared between two or three strains (Fig. 4 and S3). These results show that heterozygosity can be used to both filter out chimeric observations, and enable improved quantification of strains.

## Attempted optimisation of DADA2 parameters

As our amplicon sequencing data contained various errors (see section *Identification of sources of amplicon errors of barcodes in S. marinoi* above), we attempted to denoise the data using DADA2 version 1.16.0 (Callahan et al. 2016). This pipeline was applied to the 43 amplicon sequencing samples of the *Sm_C12W1* barcode locus, from mixtures of strains with various strain complexity (same data as shown in Fig. 4 and S3). The results of this analysis are presented in Table S7.

All samples were first quality controlled using Cutadapt version 3.2 (Martin 2011), as described in the Materials and Methods section of the main text, cutting the barcode length from 523 bp to 484 bp with primer sequences removed. To establish a baseline against which to compare the performance of DADA2, we merged the paired-end reads using BBMerge version 38.86 (default parameters) (Bushnell et al. 2017), with no denoising (Row **Exact matches** in Table S6). While this returned all 110 expected *Sm_C12W1* alleles, it also returned 149,753 false positives (defined here as those ASVs with a length +/- 12 bp the expected 484 bp barcode length, and containing 5’ and 3’ regions identical to the expected alleles, thus filtering out truncated amplicons, off-targets, and non-*Sm_C12W1* amplicons).

We next attempted to run the reads through the nf-core/ampliseq pipeline version 2.4.0 (Straub et al. 2020), which includes a DADA2 step for denoising and read merging (**ampliseq**). However, this returned none of the *Sm_C12W1* alleles. Upon closer inspection, this was due to the minOverlap and maxMismatch parameters of DADA2’s mergePairs function being set to the default values (12 and 0, respectively). These parameters are not adjustable within the nf-core/ampliseq pipeline. Thus, we attempted to run DADA2 as a standalone program, keeping many of the parameter values used by nf-core/ampliseq, but adjusting the mergePairs parameter values to match the BBMerge defaults (minOverlap 0 and maxMismatch 20), as well as setting removeBimeraDenovo’s minFoldParentOverAbundance parameter to 90 (default 1.5) (**DADA2 with merging**). While this returned all but one of the expected alleles, we also obtained almost a thousand false positives, as well as experiencing unexpected drop-out of several expected alleles in some samples.

Despite attempts to solve these issues within DADA2, we ultimately opted to merge the reads using BBMerge, before passing the merged reads to DADA2 as ‘single-ended’ reads. In addition to running the previous iteration of the pipeline on these pre-merged reads (**Relaxed DADA2 on amplicons**), we also ran the pipeline with DADA2 default settings, but implementing an error inflation step to compensate for the adjustment of quality scores in the read overlaps as performed by BBMerge (this additional step is recommended by one of DADA2’s developers on the software’s GitHub page) (**Stringent DADA2 on amplicons**).

# Supplemental Tables and Figures

Table S1: Genetic variability amongst 55 *Skeletonema marinoi* strains in common metabarcoding loci previously suggested as having some degree of intraspecific resolution in the diatom order *Thalassiosirales*. Abbreviations – ITS: internally transcribed spacer; rbcL: ribulose-1,5-bisphosphate carboxylase/oxygenase large subunit; COI: cytochrome *c-*oxidase subunit 1.

| Gene | Coordinates | Length (bp) | SNPs | Strains with same genotypes (max 55) |
| --- | --- | --- | --- | --- |
| 18S rRNA | Sm_000009F:1494606-1496397 | 1700 | 1 | 52 |
| 28S rRNA^†^ | Sm_000009F:1497116-1497900 | 785 | 0 | 55 |
| (18S+ITS1+5.8S+ITS2) ^‡^ | Sm_000009F:1496358-1497139 | 800 | 8 | 46 |
| rbcL | Sm_plastid:99162-100634 | 1473 | 0 | 55 |
| COI^§^ | Sm_mitochondrion:2060-3562 | 1503 | 2 | 53 |

^†^ Used to resolve abundance of species within the *Skeletonema* genus using metabarcoding (Canesi and Rynearson 2016)

^‡^ Partial. Proposed as an intraspecific or population marker loci for *Thalassiosirales* by Guo et al. (2015)

^§^ Proposed to have some intraspecific variation in *Skeletonema* genus by Yamada et al. (2017)

Table S2: Strains of *Skeletonema marinoi* used in this paper. The isolation date refers to when the strain was isolated from resting stages in sediment samples. Growth rate is as measured immediately before strains were mixed for the selection experiment. Start density refers to the microscopic counts of cells added to the selection experiment, and the relative start density refers to the fraction of the total population made up by cells of that strain. Relative start density (barcode) refers to the fraction of the total population made up by cells of that strain, based on proportions of ASV counts of the *Sm_C12W1* locus at the start of the experiment.

| Strain | Accession no. (genotyping) | Accession no. (amplicons) | Isolation date | Growth rate (day^-1^) | Start density (cells mL^-1^) | Relative start density (% cells of total) | Relative start density (barcode) |
| --- | --- | --- | --- | --- | --- | --- | --- |
| **Gropviken** | | | | | | | |
| GP2-4_26 | SRR23684758 | SRR23684687 | 9/28/18 | 1.41 | 21.3 | 4.10 | 3.55 |
| GP2-4_27 | SRR23684757 | SRR23684686 | 9/28/18 | 1.53 | 17.9 | 3.43 | 2.14 |
| GP2-4_28 | SRR23684642 | SRR23684685 | 9/28/18 | 1.50 | 12.6 | 2.41 | 2.93 |
| GP2-4_29 | SRR23684624 | SRR23684684 | 9/28/18 | 1.30 | 25.6 | 4.92 | 2.50 |
| GP2-4_31 | SRR23684613 | SRR23684682 | 9/28/18 | 1.15 | 17.1 | 3.27 | 3.62 |
| GP2-4_32 | SRR23684694 | - | 9/28/18 | 1.17 | 12.9 | 2.47 | 2.15 |
| GP2-4_39 | SRR23684683 | SRR23684680 | 9/28/18 | 1.48 | 9.87 | 1.89 | 1.43 |
| GP2-4_40 | SRR23684672 | SRR23684679 | 9/28/18 | 1.32 | 16.0 | 3.07 | 2.68 |
| GP2-4_42 | SRR23684780 | SRR23684677 | 9/28/18 | 0.0630 | 23.3 | 4.47 | 3.55 |
| GP2-4_43** | SRR23684769 | SRR23684676 | 9/28/18 | 1.52 | 17.4 | 3.34 | 4.05 |
| GP2-4_44 | SRR23684756 | SRR23684675 | 9/28/18 | 1.31 | 32.7 | 6.27 | 3.44 |
| GP2-4_45 | SRR23684745 | SRR23684674 | 9/28/18 | 1.43 | 25.3 | 4.85 | 5.86^†^ |
| GP2-4_46 | SRR23684734 | SRR23684673 | 9/28/18 | 1.60 | 19.5 | 3.74 |  |
| GP2-4_47 | SRR23684723 | SRR23684671 | 9/28/18 | 1.54 | 14.5 | 2.79 | 3.82 |
| GP2-4_51 | SRR23684712 | SRR23684670 | 9/28/18 | 0.783 | 21.9 | 4.20 | 4.57 |
| GP2-4_52 | SRR23684701 | SRR23684669 | 9/28/18 | 1.51 | 29.5 | 5.66 | 4.27 |
| GP2-4_54* | SRR23684805 | SRR23684667 | 9/28/18 | 1.26 | 14.8 | 2.84 | 3.33 |
| GP2-4_55 | SRR23684794 | SRR23684786 | 9/28/18 | 1.40 | 13.3 | 2.55 | 2.08 |
| GP2-4_56 | SRR23684664 | SRR23684785 | 9/28/18 | 1.11 | 15.6 | 3.00 | 3.33 |
| GP2-4_57 | SRR23684653 | SRR23684784 | 9/28/18 | 1.28 | 28.5 | 5.47 | 7.66 |
| GP2-4_61 | SRR23684641 | SRR23684783 | 9/28/18 | 1.49 | 14.6 | 2.79 | 2.16 |
| GP2-4_63** | SRR23684633 | SRR23684782 | 9/28/18 | 1.55 | 17.1 | 3.27 | 10.95 |
| GP2-4_67 | SRR23684632 | SRR23684781 | 9/28/18 | 1.55 | 16.1 | 3.08 | 4.28 |
| GP2-4_68 | SRR23684631 | SRR23684779 | 9/28/18 | 1.41 | 18.2 | 3.50 | 2.70 |
| GP2-4_69 | SRR23684630 | SRR23684778 | 9/28/18 | 1.52 | 15.7 | 3.02 | 2.74 |
| GP2-4_70 | SRR23684629 | SRR23684777 | 9/28/18 | 1.46 | 12.8 | 2.45 | 1.63 |
| GP2-4_71 | SRR23684628 | SRR23684681 | 9/28/18 | 0.778 | 22.1 | 4.25 | 4.96 |
| GP2-4_72 | SRR23684627 | SRR23684678 | 9/28/18 | 1.58 | 15.2 | 2.91 | 3.28 |
| **Gäsfjärden** | | | | | | | |
| VG1-2_44 | SRR23684626 | SRR23684776 | 1/14/19 | 1.58 | 12.0 | 2.89 | 5.24 |
| VG1-2_45** | SRR23684625 | SRR23684775 | 1/14/19 | 1.33 | 26.0 | 6.25 | 8.35 |
| VG1-2_49 | SRR23684623 | SRR23684774 | 1/14/19 | 1.48 | 10.7 | 2.57 | 2.29 |
| VG1-2_56** | SRR23684622 | SRR23684773 | 1/14/19 | 1.63 | 18.7 | 4.48 | 1.33 |
| VG1-2_58 | SRR23684621 | SRR23684772 | 1/14/19 | 1.52 | 13.1 | 3.14 | 1.51 |
| VG1-2_61** | SRR23684620 | SRR23684771 | 1/14/19 | 1.61 | 12.8 | 3.07 | 3.88 |
| VG1-2_63 | SRR23684619 | SRR23684770 | 1/14/19 | 0.569 | 17.3 | 4.14 | 2.28 |
| VG1-2_64 | SRR23684618 | SRR23684768 | 1/14/19 | 1.58 | 19.5 | 4.68 | 1.95 |
| VG1-2_65 | - | - | 1/14/19 | 1.44 | 12.3 | 2.95 | 1.73/2.10^‡^ |
| VG1-2_66 | SRR23684617 | SRR23684767 | 1/14/19 | 1.37 | 11.8 | 2.82 | 6.93 |
| VG1-2_67 | SRR23684616 | SRR23684766 | 1/14/19 | 1.68 | 18.8 | 4.52 | 1.39 |
| VG1-2_72 | SRR23684615 | SRR23684765 | 1/14/19 | 1.74 | 12.5 | 2.99 | 2.90 |
| VG1-2_74 | SRR23684614 | SRR23684764 | 1/14/19 | 1.89 | 11.2 | 2.68 | 2.49 |
| VG1-2_78* | SRR23684612 | SRR23684763 | 1/14/19 | 1.85 | 11.6 | 2.78 | 2.81 |
| VG1-2_81 | SRR23684611 | SRR23684762 | 1/14/19 | 1.78 | 8.67 | 2.08 | 5.73 |
| VG1-2_83 | SRR23684610 | SRR23684761 | 1/14/19 | 1.68 | 19.0 | 4.56 | 2.78 |
| VG1-2_85 | SRR23684609 | SRR23684760 | 1/14/19 | 1.51 | 14.0 | 3.37 | 1.63 |
| VG1-2_86 | SRR23684608 | SRR23684759 | 1/14/19 | 1.54 | 12.5 | 3.00 | 3.50 |
| VG1-2_88 | SRR23684700 | SRR23684755 | 1/14/19 | 1.46 | 12.3 | 2.95 | 3.19 |
| VG1-2_89 | SRR23684698 | SRR23684754 | 1/14/19 | 0.698 | 19.4 | 4.66 | 1.79 |
| VG1-2_90 | SRR23684697 | SRR23684753 | 1/14/19 | 1.53 | 11.8 | 2.83 | 14.46 |
| VG1-2_93 | SRR23684696 | SRR23684752 | 1/14/19 | 1.36 | 10.5 | 2.52 | 3.07 |
| VG1-2_94 | SRR23684695 | SRR23684751 | 1/14/19 | 1.43 | 10.8 | 2.58 | 4.98 |
| VG1-2_95 | SRR23684693 | SRR23684750 | 1/14/19 | 1.42 | 14.8 | 3.54 | 2.28 |
| VG1-2_99 | - | - | 1/14/19 | 1.59 | 8.58 | 2.06 | 1.73/2.10^‡^ |
| VG1-2_103 | SRR23684692 | SRR23684749 | 1/14/19 | 1.86 | 14.0 | 3.36 | 0.78 |
| VG1-2_104 | SRR23684691 | SRR23684748 | 1/14/19 | 1.58 | 12.8 | 3.07 | 4.68 |
| VG1-2_105 | SRR23684690 | SRR23684747 | 1/14/19 | 1.55 | 13.2 | 3.17 | 0.94 |
| VG1-2_106 | SRR23684689 | SRR23684746 | 1/14/19 | 1.32 | 14.0 | 3.36 | 1.46 |
| VG1-2_107** | SRR23684688 | SRR23684744 | 1/14/19 | 1.72 | 11.8 | 2.84 | 1.46 |
| * Triploid strains (otherwise strains are assumed to be diploid)  ** Strains displaying skewed allelic ratios (based on both the single strain genotyping amplicons and the selection experiment [Fig. 4])  ^†^ As strains GP2-4_45 and GP2-4_46 were found to be clones, their relative start density (barcode) is based on their combined proportion of ASV counts  ^‡^ As strains VG1-2_65 and VG1-2_99 did not survive antibiotic treatment, unassigned high frequency ASVs were assumed to belong to them, and were used to estimate their relative start densities | | | | | | | |

Table S3: Strains of *Chlamydomonas reinhardtii* used in this paper, their accession numbers, the database from which they were retrieved (either NCBI’s Short Read Archive [SRA], or the European Nucleotide Archive [ENA]), and the relevant citation.

| **Strain** | **Accession no.** | **Database** | **Citation** |
| --- | --- | --- | --- |
| **Mating type +** | | | |
| CC-2343 | SRX823807 | SRA | (Flowers et al. 2015) |
| CC-2344 | SRX823856 | SRA | (Flowers et al. 2015) |
| CC-2936 | SRX823810 | SRA | (Flowers et al. 2015) |
| CC-2937 | SRX823858 | SRA | (Flowers et al. 2015) |
| CC-3065 | SAMEA4731376 | ENA | (Ness et al. 2016) |
| CC-3071 | SAMEA4731378 | ENA | (Ness et al. 2016) |
| CC-3076 | SAMEA4731381 | ENA | (Ness et al. 2016) |
| CC-3086 | SAMEA4731384 | ENA | (Ness et al. 2016) |
| **Mating type -** | | | |
| CC-2290 | SRX823806 | SRA | (Flowers et al. 2015) |
| CC-2342 | SRX823805 | SRA | (Flowers et al. 2015) |
| CC-2931 | SRX823857 | SRA | (Flowers et al. 2015) |
| CC-2935 | SRX823809 | SRA | (Flowers et al. 2015) |
| CC-2938 | SRX823808 | SRA | (Flowers et al. 2015) |
| CC-3059 | SAMEA4731370 | ENA | (Ness et al. 2016) |
| CC-3063 | SAMEA4731374 | ENA | (Ness et al. 2016) |
| CC-3079 | SAMEA4731382 | ENA | (Ness et al. 2016) |
| CC-3084 | SAMEA4731383 | ENA | (Ness et al. 2016) |

Table S4: Oligo primers used or developed in this study.

| Taxa designed for | Name | Sequence (5'-->3') |
| --- | --- | --- |
| Eukaryotes (18S rRNA gene) ^†^ | TAReuk454FWD1 | CCAGCASCYGCGGTAATTCC |
| Eukaryotes (18S rRNA gene) ^†^ | TAReuk454REV3 | ACTTTCGTTCTTGATYRA |
| Prokaryotes (16S rRNA gene) ^‡^ | 341F | CCTAYGGGRBGCASCAG |
| Prokaryotes (16S rRNA gene) ^‡^ | 806R | GGACTACNNGGGTATCTAAT |
| Diatoms ^§^ | rbcL-F | ATGTCTCAATCTGTAWCAGAACGGACTC |
| *Skeletonema marinoi/subsalsum* | rbcL-R | GATACCTGTAGCAGGACCTTGG |
| *Skeletonema marinoi* | Sm_C2W24-F | CATGAAACGGAAACTCTGAGCAC |
| *Skeletonema marinoi* | Sm_C2W24-R | AATATGCTGCGTTTCGAGTTCAATG |
| *Skeletonema marinoi* | Sm_C12W1-F | AGGYTTCGCCTCCTCAAAC |
| *Skeletonema marinoi* | Sm_C12W1-R | GGCACGATGCACACGCAAAG |
| *Skeletonema marinoi* | Sm_C12W2-F | CCCTCCAATACGCAACAATCC |
| *Skeletonema marinoi* | Sm_C12W2-R | CAATTCCACACGTCCCACC |
| *Skeletonema marinoi* | Sm_C16W4-F | CTGGTCCYATCAAGTGTGTGTCATC |
| *Skeletonema marinoi* | Sm_C16W4-R | TTCCCGTGAGGAGAGTGGAWG |
| *Chlorophytes* | Chlamy18SF | TGCCCTATCAACTTTCGATGGT |
| *Chlorophytes* | Chlamy18SR | GTGTGTACAAAGGGCAGGGA |
| *Chlamydomonas reinhardtii (mt-)* | Cr_Chr9W12-F | GTCACACTGCCCTGGTC |
| *Chlamydomonas reinhardtii(mt-)* | Cr_Chr9W12-R | CACCCACCTGTGCGATA |
| *Chlamydomonas reinhardtii(mt+)* | Cr_Chr1W3-F | TCGCTGCCACACTTCAC |
| *Chlamydomonas reinhardtii(mt+)* | Cr_Chr1W3-R | GGTTGGTCCAGCAGTCAG |
| *Chlamydomonas reinhardtii(mt+)* | Cr_Chr3W8-F | CTCCACATGGCCCTTAG |
| *Chlamydomonas reinhardtii(mt+)* | Cr_Chr3W8-R | CTGGTGAACCGGAATGT |
| *Chlamydomonas reinhardtii(mt+)* | Cr_Chr3W10-F | CAACGCGGCTGTTGGTC |
| *Chlamydomonas reinhardtii(mt+)* | Cr_Chr3W10-R | CGATCGGAGGTTTATATGCGTTTC |
| *-* | *Illumina F adapter* | ACACTCTTTCCCTACACGACGCTCTTCCGATCT |
| *-* | *Illumina R adapter* | GTGACTGGAGTTCAGACGTGTGCTCTTCCGATCT |

^†^(Stoeck et al. 2010), ^‡^(Sundberg et al. 2013), ^§^(Guo et al. 2015)

Table S5: List of all 26 barcoding loci proposed by Bamboozle, with the genomic coordinates, the sequence of the conserved regions, and the putative function of the gene containing the locus.

| Species | ID | Genomic coordinates | Total length (bp) | 5' end conserved | 3' end conserved | Gene model (annotation) |
| --- | --- | --- | --- | --- | --- | --- |
| *S. marinoi* | *Sm_C12W1* | Sm_000012F: 1009438..1009953 | 516 | CCTCAAACCCCATCGAATACAAACTCTCCATATGCA | GCTTTGCGGAATCTTTGCGTGTGCATCGTGCCCATG | QTG54_008021 (TPR-like superfamily protein) |
| *S. marinoi* | *Sm_C12W2* | Sm_000012F: 1011059..1011599 | 541 | GAACTCCAAAAAACACCCCTCCAATACGCAACAATCCCCGCAGTAGCCGCCTTCCTAGGCC | TTGTTTCAAAAGGTGGGACGTGTGGAATTGGATTTTTTGGTGGAAAGTGGATTGGGGTTTG | QTG54_008022 (Uncharacterised membrane protein) |
| *S. marinoi* | *Sm_C12W3* | Sm_000012F: 1011134..1011634 | 501 | GCCGGAGTCAAAATGTTATTC | TTGGTGCAGATGGTGGCGTGG | QTG54_008022 (Uncharacterised membrane protein) |
| *S. marinoi* | *Sm_C16W4* | Sm_000016F: 654027..654528 | 502 | ATCAAGTGTGTGTCATCTTTGA | ACTCTCCTCACGGGAAGTTGGG | QTG54_009562 (ribosome biogenesis protein WDR12) |
| *C. reinhardtii* (mt-) | *Cr_Chr2W4* | chromosome_2: 5974497-5974828 | 332 | GCGCCGGCCCTGACTCTATCTTTTGAAAGTTCACGAGATGTAGGCAAAGAGC | CGCTGGCGGACGCGCTCAGCCGGAGCCTGCGGGAGCGCTACCCCGAAAGCAA | CHLRE_02g112150v5 (phospholipase B) |
| *C. reinhardtii* (mt-) | *Cr_Chr9W12* | chromosome_9: 3429678-3429981 | 304 | CACCACGTCACACTGCCCTGGTCC | ATCGCACAGGTGGGTGCCGACGCG | CHLRE_09g389134v5 (unknown function (DUF563)) |
| *C. reinhardtii* (mt-) | *Cr_Chr9W13* | chromosome_9: 3429715-3430015 | 301 | ACCTGGTGTCCATCCTGCAGG | CAGCTTACAAGGCGGGCGTGC | CHLRE_09g389134v5 (unknown function (DUF563)) |
| *C. reinhardtii* (mt-) | *Cr_Chr10W15* | chromosome_10: 3895695-3895996 | 302 | GCGGCAACCGGTGCCCAGCGCG | CGTGGCCAAAGCGGTGCCGGCG | CHLRE_10g447800v5 (60kDa SS-A/Ro ribonucleoprotein?) |
| *C. reinhardtii* (mt-) | *Cr_Chr13W19* | chromosome_13: 4163058-4163376 | 319 | GTGCGGCCGGGCGCGGGTAAGTGCCTGGCTGAGGTCGAC | GCGTCATCGACGTGCACATCCACCTCAATGAGCCCGGTC | CHLRE_13g592050v5 (allantoinase) |
| *C. reinhardtii* (mt-) | *Cr_Chr16W20* | chromosome_16: 2930253-2930565 | 313 | TGCCACGCCGGATGCCATACACAGCATGCGCCC | TGAAGCTGCTGCCGGACGCCAAGTGCGTGAGCG | CHLRE_16g664050v5 (GRAM domain? DUF4782?) |
| *C. reinhardtii* (mt-) | *Cr_Chr17W6* | chromosome_17: 1200452-1200762 | 311 | TATATGGCGCAGGAGGTGACGGTGGTCCAGG | CAGGCCAACGCCAAGCGCGACCGTGATGTGG | CHLRE_17g704950v5 (unknown function) |
| *C. reinhardtii* (mt+) | *Cr_Chr1W1* | chromosome_1: 5482222-5482550 | 329 | GACCGTGACTACGGCATCTTCAACAAGATCCACCACGACATCGGCACCC | CTGCACAGATTCCCCACTACAACCTTGAGGAGGCTACCGAGGCCGTCAA | CHLRE_01g038600v5 (chloroplast glycerolipid omega-3-fatty acid desaturase) |
| *C. reinhardtii* (mt+) | *Cr_Chr1W2* | chromosome_1: 5482306-5482615 | 310 | AGGGCTGAATCTGGGTCGGGTTGGGAAATG | AGAGCCCCGGCCCCCTGCCCACCCACCTGG | CHLRE_01g038600v5 (chloroplast glycerolipid omega-3-fatty acid desaturase) |
| *C. reinhardtii* (mt+) | *Cr_Chr1W3* | chromosome_1: 5735287-5735592 | 306 | GGTCGCTGCCACACTTCACCCCGAGG | TCCTGACTGCTGGACCAACCGCTCGC | CHLRE_01g040900v5 (unknown function) |
| *C. reinhardtii* (mt+) | *Cr_Chr3W5* | chromosome_3: 3981328-3981632 | 305 | CCACCTACCCCTCCCGCTCCGTGTC | CACAGACGAGGAGCGGGAGGAGGAG | CHLRE_03g171850v5 (unknown function) |
| *C. reinhardtii* (mt+) | *Cr_Chr3W6* | chromosome_3: 5068965-5069267 | 303 | ACATGAGCGTTGCAACATGTGTA | GCTATGCTGTCCGTCGGTTTTTG | CHLRE_03g181050v5 (unknown function) |
| *C. reinhardtii* (mt+) | *Cr_Chr3W7* | chromosome_3: 5075944-5076245 | 302 | TGGTTCACGGGCGGCACCGTGA | GCAGCGTAGTTTTGATATGGGC | CHLRE_03g181100v5 (hypothetical protein) |
| *C. reinhardtii* (mt+) | *Cr_Chr3W8* | chromosome_3: 5075977-5076278 | 302 | TCCTCCACATGGCCCTTAGCCT | GCACATTCCGGTTCACCAGGTC | CHLRE_03g181100v5 (hypothetical protein) |
| *C. reinhardtii* (mt+) | *Cr_Chr3W9* | chromosome_3: 6202804-6203115 | 312 | CGGCCGCACCTTGCCCGCCACCGCGGCGCCGC | ATCCTGGCCAAGTGGCTGGCTTGCAAGGCTGC | CHLRE_03g192350v5 (Kelch motif?) |
| *C. reinhardtii* (mt+) | *Cr_Chr3W10* | chromosome_3: 7273555-7273862 | 308 | GCACGCAACGCGGCTGTTGGTCTCGCTG | GAAACGCATATAAACCTCCGATCGAATG | CHLRE_03g207250v5 (putative glutamine synthetase) |
| *C. reinhardtii* (mt+) | *Cr_Chr6W11* | chromosome_6: 7174112-7174428 | 317 | GAGGTCAGGGTAGCTGCTGTGGCCTCGGACTTGAGGG | CCTTGGCGGTCAGCGTCATGTGCCCGGTTGGAAGCCC | CHLRE_06g297800v5 (hypothetical protein) |
| *C. reinhardtii* (mt+) | *Cr_Chr11W16* | chromosome_11: 59098-59403 | 306 | AGATGTTGATGTTGATGGCGCCGAAC | TGTTGATGGACCTCAGCGGCCGCAGC | CHLRE_11g467528v5 (calcium channel) |
| *C. reinhardtii* (mt+) | *Cr_Chr11W17* | chromosome_11: 59130-59458 | 329 | AAGTAGAACACGGCCAGGATGACCACGTCCAGCAGCAGCGGAATGGAGT | ACGCAGCGGATGAGTGTGTAGTTGCCGCGGCCCGACAGATCCAGGTAGC | CHLRE_11g467528v5 (calcium channel) |
| *C. reinhardtii* (mt+) | *Cr_Chr11W18* | chromosome_11: 59181-59493 | 313 | AGCATGGTGTCCACCAGTTGCTGTGTGGGGTAA | AGGCCCACAACCACAAAGTCTATGATGTTCCAC | CHLRE_11g467528v5 (calcium channel) |
| *C. reinhardtii* (mt+) | *Cr_Chr17W21* | chromosome_17: 1192804-1193104 | 301 | TGCCCTGGATCTTGTCGCGGC | AAGTGCACTGCACCGTGTGTG | CHLRE_17g704850v5 (adenine phosphoribosyltransferase) |
| *C. reinhardtii* (mt+) | *Cr_Chr17W22* | chromosome_17: 1192828-1193181 | 354 | TGAGGAAGGGCAGCTCGATGACGCACGCCGCCTCCACCACCACACCGCCGGCCTTCTCTGTGGGGCGGTGAGGG | GGCGCATGTGAGACTTACTGACGAGGTTGATGCCGGCGGCAAGGGTGCCGCCGGTGGCAATCAGGTCATCAACC | CHLRE_17g704850v5 (adenine phosphoribosyltransferase) |

Table S6: Primer mismatches in other populations of *S. marinoi* and species within the order *Thalassiosirales*. Sequences were obtained from either the Marine Microbial Eukaryote Transcriptome Sequencing Project (*S. marinoi* [Keeling et al. 2014]), NCBI (*Thalassiosira pseudonana* [Armbrust et al. 2004] and *Thalassiosira oceanica* [Lommer et al. 2012]), or an in-house draft genome assembly (*Skeletonema subsalsum*). NA indicates that the genomic region of the barcode could not be identified using BLASTn, and ND indicates insufficient sequencing depth of transcriptomic data (<1 coverage across the primer site) to determine if mismatches were present. Gene model homologs in *Thalassiosira* included – *Sm_ C2W24*: NA; *Sm_C12W1*: THAPS3_24049; *Sm_C12W2*: THAPS3_5794, THAOCEA1_01345; *Sm_C16W4*: THAPS3_22985, THAOCEA1_70515.

| Primer site | Adriatic Sea *S. marinoi* (strain FE7) | Adriatic Sea *S. marinoi* (strain FE60) | Narragansett Bay *S. marinoi* (strain skelA) | *S. subsalsum* (strain LO-03-75) | *T. pseudonana* (strain CCMP1335) | *T. oceanica* (strain CCMP1005) |
| --- | --- | --- | --- | --- | --- | --- |
| *Sm_C2W24-F* | 0 | 0 | 0 | NA | NA | NA |
| *Sm_C2W24-R* | 0 | 0 | 0 | NA | NA | NA |
| *Sm_C12W1-F* | 0 | 0 | 0 | 4 | 3 | NA |
| *Sm_C12W1-R* | 0 | 0 | 0 | 0 | 7 | NA |
| *Sm_C12W2-F* | 0 | ND | 0 | 1 | 3 | >10 |
| *Sm_C12W2-R* | 0 | 0 | 0 | 0 | 4 | >10 |
| *Sm_C16W4-F* | ND | ND | 0 | 5 | 7 | 7 |
| *Sm_C16W4-R* | ND | ND | 0 | 3 | 6 | 7 |

Table S7: Optimisation of the denoising pipeline for amplicon sequencing data, based on experimental observations. Results derived from analysis of amplicon sequence datasets from three timepoints (4-5 replicates each) of the *Sm_C12W1* barcode (in total 43 datasets; 21 containing only strains from the GP population, 22 containing only strains from the VG population). Additional details on each iteration are given in the *Attempted optimisation of DADA2 parameters* section of the Supplemental Information.

| **Iteration** | Total ASVs^1^ | False negative ASVs^2^ | False positive ASVs^3^ | Read proportion TP^4^ | Read proportion FP^5^ | Mismatches between populations^6^ |
| --- | --- | --- | --- | --- | --- | --- |
| Exact matches (BBMerge without denoising) | 228,804 | 0 | 148,753 | 11.11% | 7.17% | 215 |
| ampliseq (integrate DADA2+Qiime2) | 1,006 | 110 | 0 | 0 | 0 | n/a |
| DADA2 with merging (DADA2) | 4,096 | 1 | 985 | 56.93% | 7.39% | 70 |
| Stringent DADA2 on amplicons (BBMerge+DADA2) | 1,062 | 3 | 16 | 17.89% | 0.03% | 1 |
| Relaxed DADA2 on amplicons (BBMerge+DADA2) | 1,483 | 0 | 346 | 17.81% | 1.19% | 4 |
| ^1^ Total ASVs: the number of unique sequences produced from merged reads, either without (*Exact matches*) or with denoising.  ^2^ False negative ASVs: the number of expected alleles that are missing from the total ASV count.  ^3^ False positive ASVs: the number of unexpected alleles that are within 12 bp of the expected 484 bp barcode length, and contain the 5’ and 3’ regions identical among the expected alleles.  ^4^ Read proportion TP: the percentage of the original read pairs that both merged successfully, and matched one of the 110 true alleles.  ^5^ Read proportion FP: the percentage of the original read pairs that both merged successfully, and matched one of the identified false positive alleles.  ^6^ Mismatches between populations: the number of ASVs expected exclusively in one population that were seen in the other population (assumed to be caused by chimeras or sequencing errors rather than cross-contamination). If all GP-exclusive alleles were observed in all VG-exclusive datasets, and vice versa, this would result in 2,149 mismatches (i.e. the maximum possible number of mismatches given these two populations). | | | | | | |

Figure S1: Relationship between microscopically determined relative abundances of cells versus barcoded relative abundances. Preserved cells from single-strain cultures were counted microscopically using a Sedgewick Rafter cell counting chamber (Wildlife Supply Company VR) (>300 cells per strain) to determine their relative abundances. Cultures were mixed at equal density based on chlorophyll fluorescence, after which a sample was collected, amplified using primers Sm_C12W1-F and Sm_C12W1-R, and subsequently sequenced. Barcoded abundances were then computed using equations 1 and 2 as described in Fig. 4. For clarity, the strain names have been shortened to the numbers only, and strains that could not be separated by the barcodes have been omitted from the analysis.


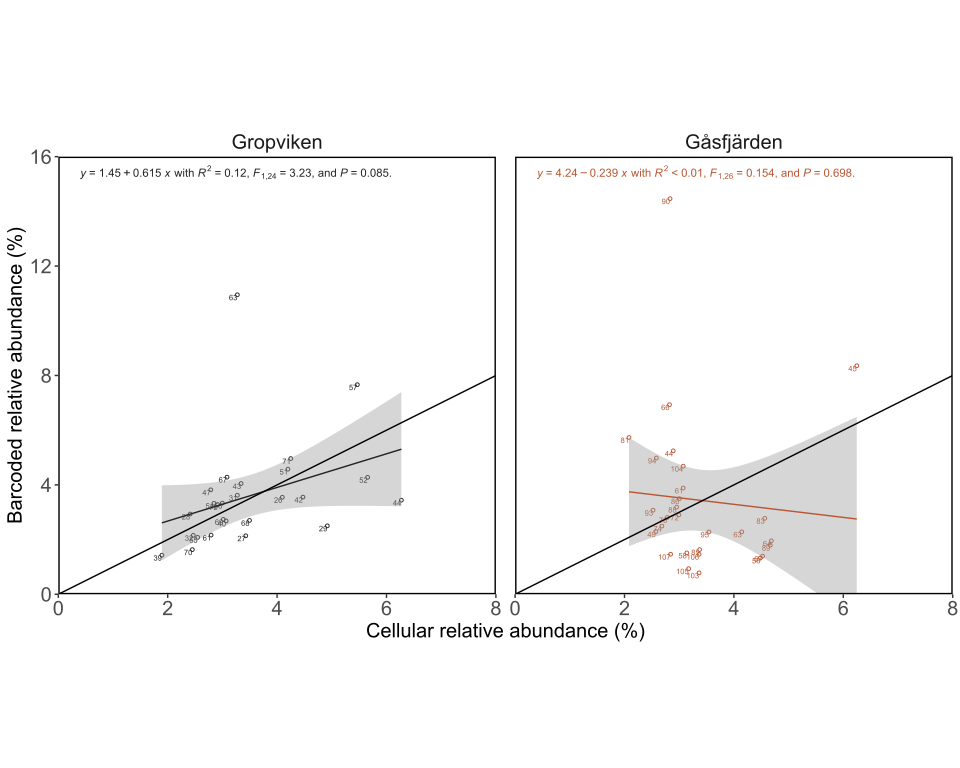


Figure S2: PCR test of species specificity in amplification of the four *S. marinoi* barcoding loci and two *C. reinhardtii* loci, using DNA from other phytoplankton. A) All four *S. marinoi* loci were assayed in the close relative *Skeletonema subsalsum*, showing that only *Sm_C12W2* amplifies efficiently in this species. B) The second PCR was performed only for *Sm_C12W1* but on a battery of microalgal samples that were mixed at equal *in vivo* chlorophyll *a* fluorescence density before DNA extraction. ‘Mix diatom’ corresponds to DNA from a mixture of seven diatom species isolated from the same Baltic Sea location as the *S. marinoi* strains (GP) and includes two different *Chaetoceros* and *Fragilariopsis* species, as well as *Thalassiosira baltica*, *Melosira* sp., and *Cyclotella* sp.. *‘*Mixture’ corresponds to DNA from a mixture of the cyanobacteria *Nodularia spumigena*, *Aphanizomenon klebahnii*, *Dolichospermum* sp., the dinoflagellate *Prorocentrum cordatum*, and a small (2-4 µm diameter) unknown coccoid chlorophyte. ‘S_marinoi’ corresponds to DNA from strain VG1-2_86 which served as a positive control for amplification of *Sm_C12W1*, and ‘NT’ refers to the negative controls. C) Lack of PCR amplification of two *Chlamydomonas reinhardtii* barcode loci in two related species, *Chlamydomonas* sp., and *Microglena* sp. Primer sequences are shown in Table S4.


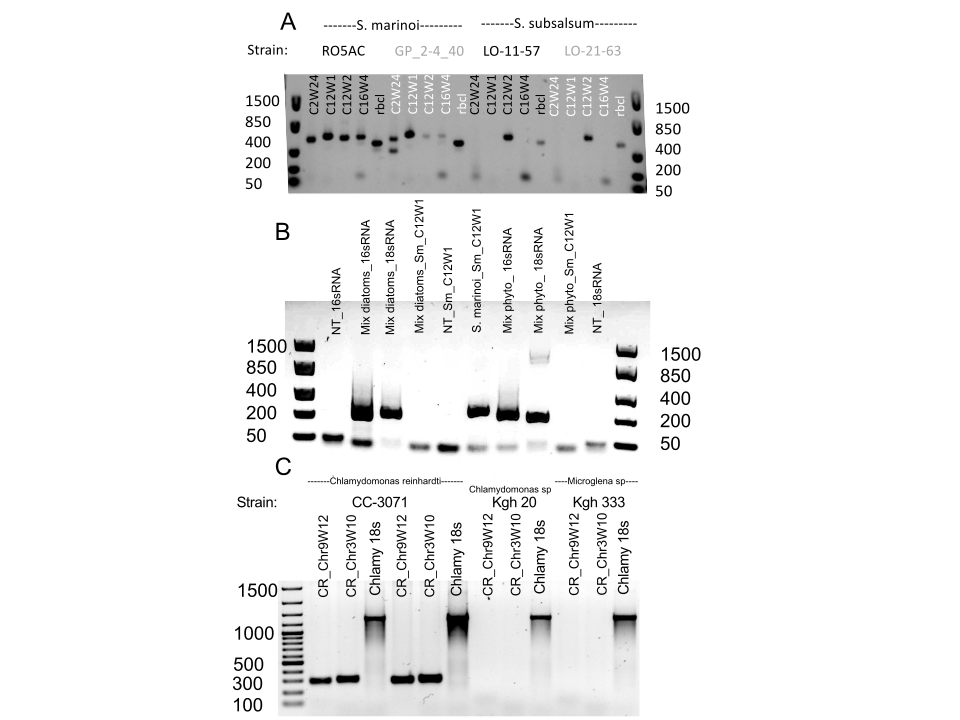


Figure S3: Correlations between allele frequencies of 58 strains of *S. marinoi* across the 42-day selection experiment. The experiment was performed separately for two populations – VG and GP. Each panel displays the allele frequencies for one strain across three timepoints (0, 9 and 42 days) and two treatments (control and copper stress). The black line across panels corresponds to the expected 1:1 ratio between alleles. The blue line (sometimes overlapped by the black line if correlation is high) is a second order polynomial function fitted to the data, with shaded area corresponding to 95% confidence interval. For visibility, data has been transformed (x +1) to allow for zero counts in one allele, and instances where neither allele from a strain was present have been removed. Alleles shared between strains are indicated by alphabetical indexes in the corner of each panel, where relevant and their counts have been assigned to the relevant strains using equations 1 and 2 described in Materials and Methods. Asterisks highlight false positive observations that the denoising pipeline failed to remove.


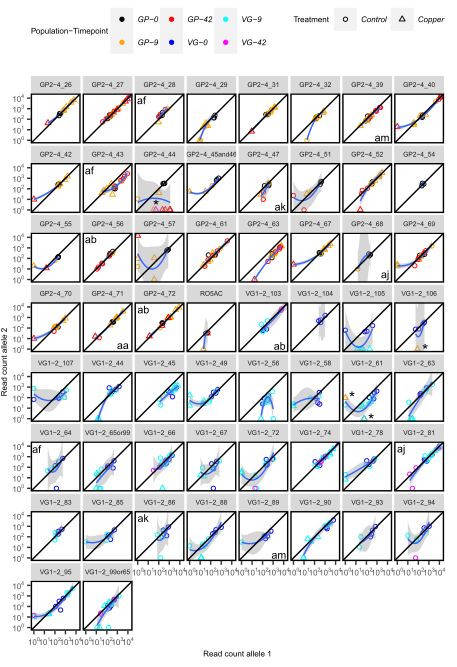


Figure S4: Pie charts depicting the breakdown of read pairs in each of the *S. marinoi* barcodes, based on the single-strain samples. Blue segments correspond to read pairs that did not merge; red segments correspond to read pairs that merged and matched the expected alleles for the given strain; and green segments correspond to unknowns, with a further breakdown of their identity in the outer ring.


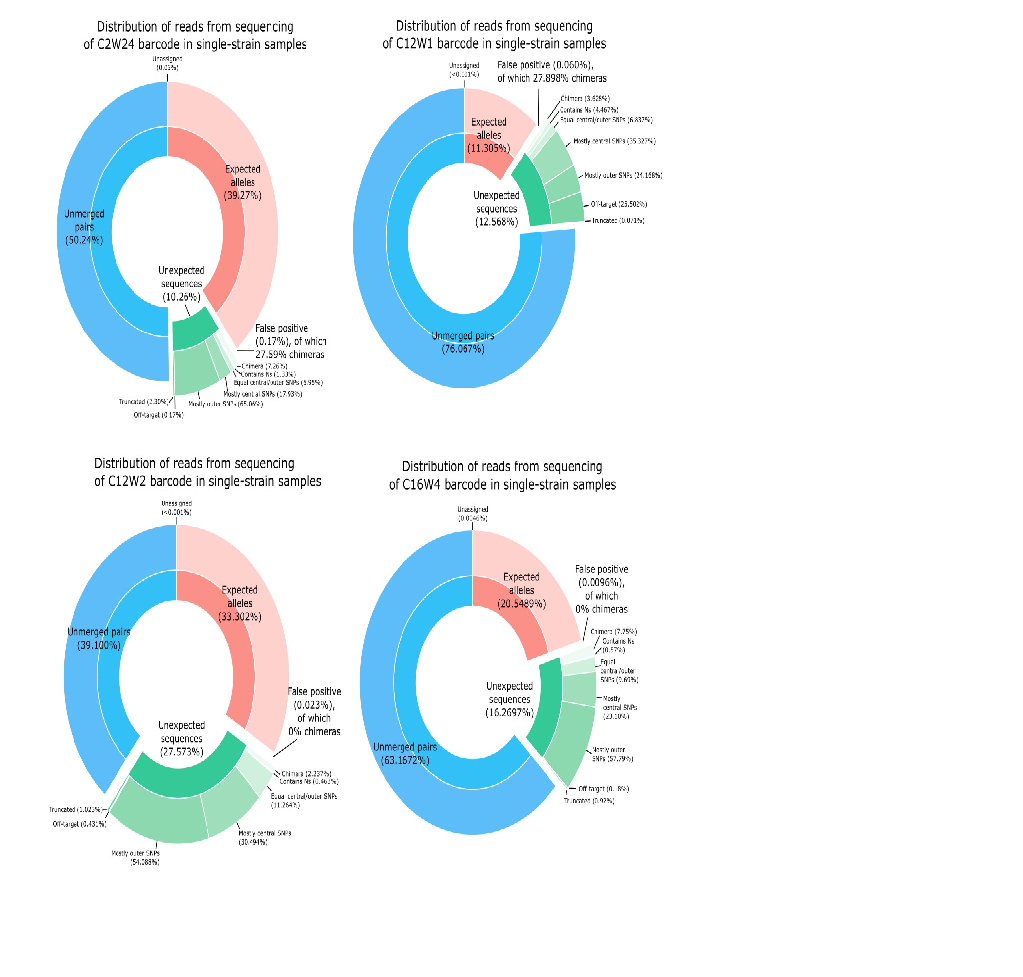


# References

Armbrust, E. V., Berges, J. A., Bowler, C., Green, B. R., Martinez, D., Putnam, N. H., Zhou, S., Allen, A. E., Apt, K. E., Bechner, M., Brzezinski, M. A., Chaal, B. K., Chiovitti, A., Davis, A. K., Demarest, M. S., Detter, J. C., Glavina, T., Goodstein, D., Hadi, M. Z., … Rokhsar, D. S. (2004). The Genome of the Diatom *Thalassiosira pseudonana*: Ecology, Evolution, and Metabolism. *Science*, *306*(5693), 79–86. https://doi.org/10.1126/science.1101156

Bushnell, B., Rood, J., & Singer, E. (2017). BBMerge – Accurate paired shotgun read merging via overlap. *PLoS One*, *12*(10), e0185056. https://doi.org/10.1371/journal.pone.0185056

Callahan, B. J., McMurdie, P. J., Rosen, M. J., Han, A. W., Johnson, A. J. A., & Holmes, S. P. (2016). DADA2: High-resolution sample inference from Illumina amplicon data. *Nature Methods*, *13*(7), 581–583. https://doi.org/10.1038/nmeth.3869

Canesi, K. L., & Rynearson, T. A. (2016). Temporal variation of *Skeletonema* community composition from a long-term time series in Narragansett Bay identified using high-throughput DNA sequencing. *Marine Ecology Progress Series*, *556*, 1–16. https://doi.org/10.3354/meps11843

Edgar, R. C. (2016). UCHIME2: improved chimera prediction for amplicon sequencing. *BioRxiv*, 74252. https://doi.org/10.1101/074252

Flowers, J. M., Hazzouri, K. M., Pham, G. M., Rosas, U., Bahmani, T., Khraiwesh, B., Nelson, D. R., Jijakli, K., Abdrabu, R., Harris, E. H., Lefebvre, P. A., Hom, E. F. Y., Salehi-Ashtiani, K., & Purugganan, M. D. (2015). Whole-Genome Resequencing Reveals Extensive Natural Variation in the Model Green Alga *Chlamydomonas reinhardtii*. *The Plant Cell*, *27*(9), 2353–2369. https://doi.org/10.1105/tpc.15.00492

Guo, L., Sui, Z., Zhang, S., Ren, Y., & Liu, Y. (2015). Comparison of potential diatom 'barcode' genes (the 18S rRNA gene and ITS, COI, *rbcL*) and their effectiveness in discriminating and determining species taxonomy in the Bacillariophyta. *International Journal of Systematic and Evolutionary Microbiology*, *65*(Pt_4), 1369–1380. https://doi.org/10.1099/ijs.0.000076

Hofmann, H., Flöss, S., & Stamminger, T. (2000). Covalent Modification of the Transactivator Protein IE2-p86 of Human Cytomegalovirus by Conjugation to the Ubiquitin-Homologous Proteins SUMO-1 and hSMT3b. *Journal of Virology*, *74*(6), 2510–2524. https://doi.org/10.1128/jvi.74.6.2510-2524.2000

Keeling, P. J., Burki, F., Wilcox, H. M., Allam, B., Allen, E. E., Amaral-Zettler, L. A., Armbrust, E. V., Archibald, J. M., Bharti, A. K., Bell, C. J., Beszteri, B., Bidle, K. D., Cameron, C. T., Campbell, L., Caron, D. A., Cattolico, R. A., Collier, J. L., Coyne, K., Davy, S. K., … Worden, A. Z. (2014). The Marine Microbial Eukaryote Transcriptome Sequencing Project (MMETSP): Illuminating the Functional Diversity of Eukaryotic Life in the Oceans through Transcriptome Sequencing. *PLoS Biology*, *12*(6), e1001889. https://doi.org/10.1371/journal.pbio.1001889

Lommer, M., Specht, M., Roy, A.-S., Kraemer, L., Andreson, R., Gutowska, M. A., Wolf, J., Bergner, S. V, Schilhabel, M. B., Klostermeier, U. C., Beiko, R. G., Rosenstiel, P., Hippler, M., & LaRoche, J. (2012). Genome and low-iron response of an oceanic diatom adapted to chronic iron limitation. *Genome Biology*, *13*(7), R66. https://doi.org/10.1186/gb-2012-13-7-r66

Martin, M. (2011). Cutadapt removes adapter sequences from high-throughput sequencing reads. *EMBnet.Journal*, *17*(1), 10–12. https://doi.org/10.14806/ej.17.1.200

Morrell, R., & Sadanandom, A. (2019). Dealing With Stress: A Review of Plant SUMO Proteases. *Frontiers in Plant Science*, *10*, 1122. https://doi.org/10.3389/fpls.2019.01122

Ness, R. W., Kraemer, S. A., Colegrave, N., & Keightley, P. D. (2016). Direct Estimate of the Spontaneous Mutation Rate Uncovers the Effects of Drift and Recombination in the *Chlamydomonas reinhardtii* Plastid Genome. *Molecular Biology and Evolution*, *33*(3), 800–808. https://doi.org/10.1093/molbev/msv272

Rognes, T., Flouri, T., Nichols, B., Quince, C., & Mahé, F. (2016). VSEARCH: a versatile open source tool for metagenomics. *PeerJ*, *4*, e2584. https://doi.org/10.7717/peerj.2584

Schloss, P. D., Westcott, S. L., Ryabin, T., Hall, J. R., Hartmann, M., Hollister, E. B., Lesniewski, R. A., Oakley, B. B., Parks, D. H., Robinson, C. J., Sahl, J. W., Stres, B., Thallinger, G. G., Van Horn, D. J., & Weber, C. F. (2009). Introducing mothur: Open-Source, Platform-Independent, Community-Supported Software for Describing and Comparing Microbial Communities. *Applied and Environmental Microbiology*, *75*(23), 7537–7541. https://doi.org/10.1128/AEM.01541-09

Stoeck, T., Bass, D., Nebel, M., Christen, R., Jones, M. D. M., Breiner, H.-W., & Richards, T. A. (2010). Multiple marker parallel tag environmental DNA sequencing reveals a highly complex eukaryotic community in marine anoxic water. *Molecular Ecology*, *19*(s1), 21–31. https://doi.org/https://doi.org/10.1111/j.1365-294X.2009.04480.x

Straub, D., Blackwell, N., Langarica-Fuentes, A., Peltzer, A., Nahnsen, S., & Kleindienst, S. (2020). Interpretations of Environmental Microbial Community Studies Are Biased by the Selected 16S rRNA (Gene) Amplicon Sequencing Pipeline. *Frontiers in Microbiology, 11*, 2652. https://doi.org/10.3389/fmicb.2020.550420

Sundberg, C., Al-Soud, W. A., Larsson, M., Alm, E., Yekta, S. S., Svensson, B. H., Sørensen, S. J., & Karlsson, A. (2013). 454 pyrosequencing analyses of bacterial and archaeal richness in 21 full-scale biogas digesters. *FEMS Microbiology Ecology*, *85*(3), 612–626. https://doi.org/10.1111/1574-6941.12148

Verger, A., Perdomo, J., & Crossley, M. (2003). Modification with SUMO: a role in transcriptional regulation. *EMBO Reports*, *4*(2), 137–142. https://doi.org/10.1038/sj.embor.embor738

Yamada, M., Otsubo, M., Tsutsumi, Y., Mizota, C., Nakamura, Y., Takahashi, K., & Iwataki, M. (2017). Utility of mitochondrial-encoded cytochrome c oxidase I gene for phylogenetic analysis and species identification of the planktonic diatom genus *Skeletonema*. *Phycological Research*, *65*(3), 217–225. https://doi.org/10.1111/pre.12179
