# Supplementary figures and images for: Bamboozle: A Bioinformatic Tool for Identification and Quantification of Intraspecific Barcodes
Source: Mol Ecol Resour. 2025 Feb 4;25(4):e14067. doi: 10.1111/1755-0998.14067 (PMC11969633; doi:10.1111/1755-0998.14067)

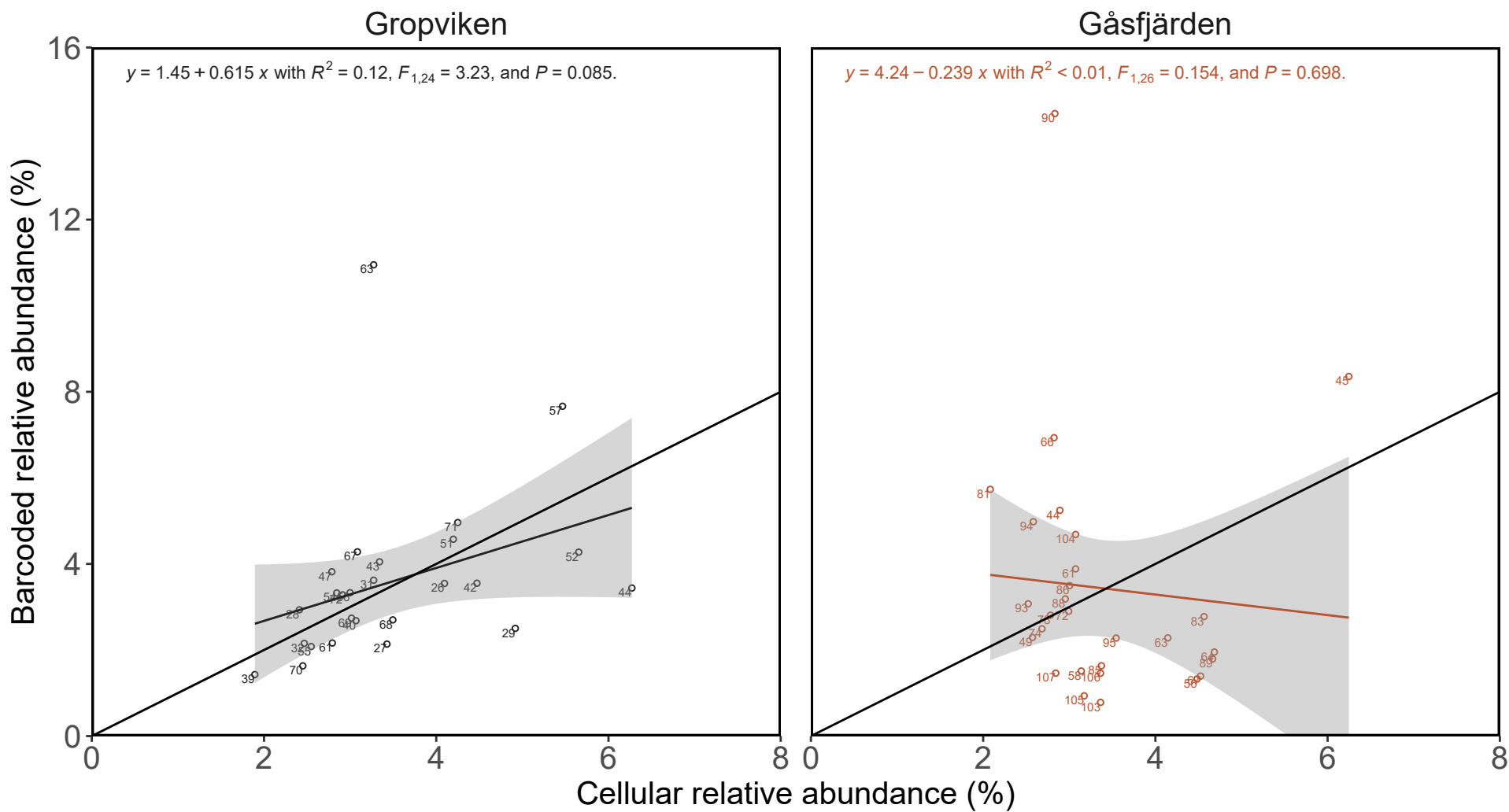

Supplement: Supplementary file 3 — Figure S1. [file MEN-25-e14067-s002.pdf]

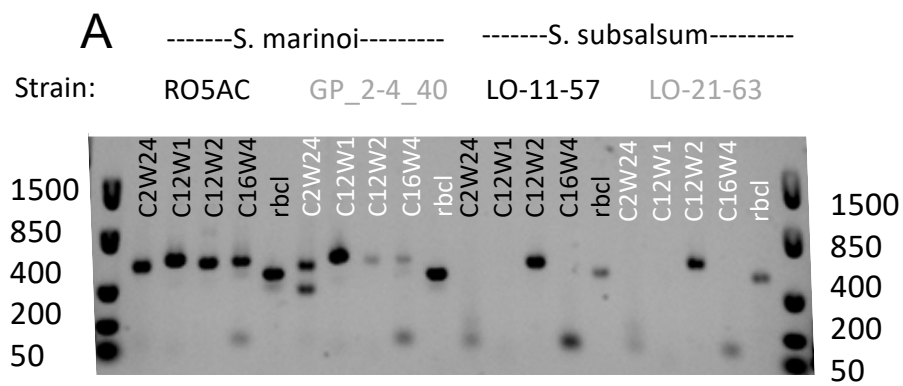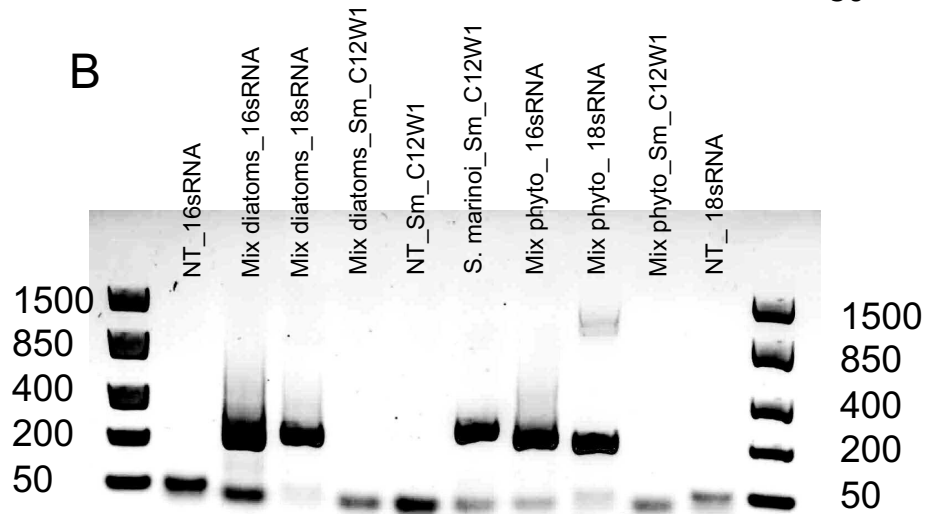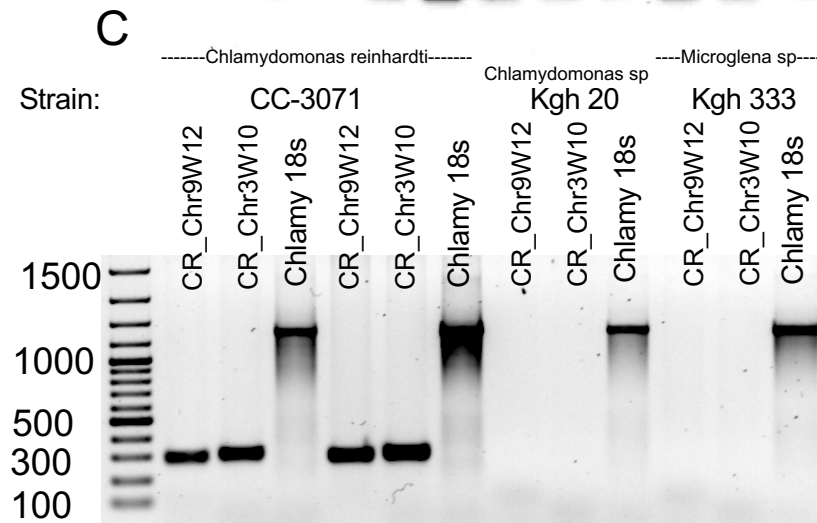

Supplement: Supplementary file 4 — Figure S2. [file MEN-25-e14067-s001.pdf]

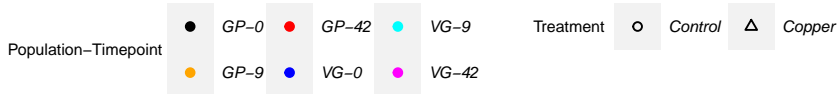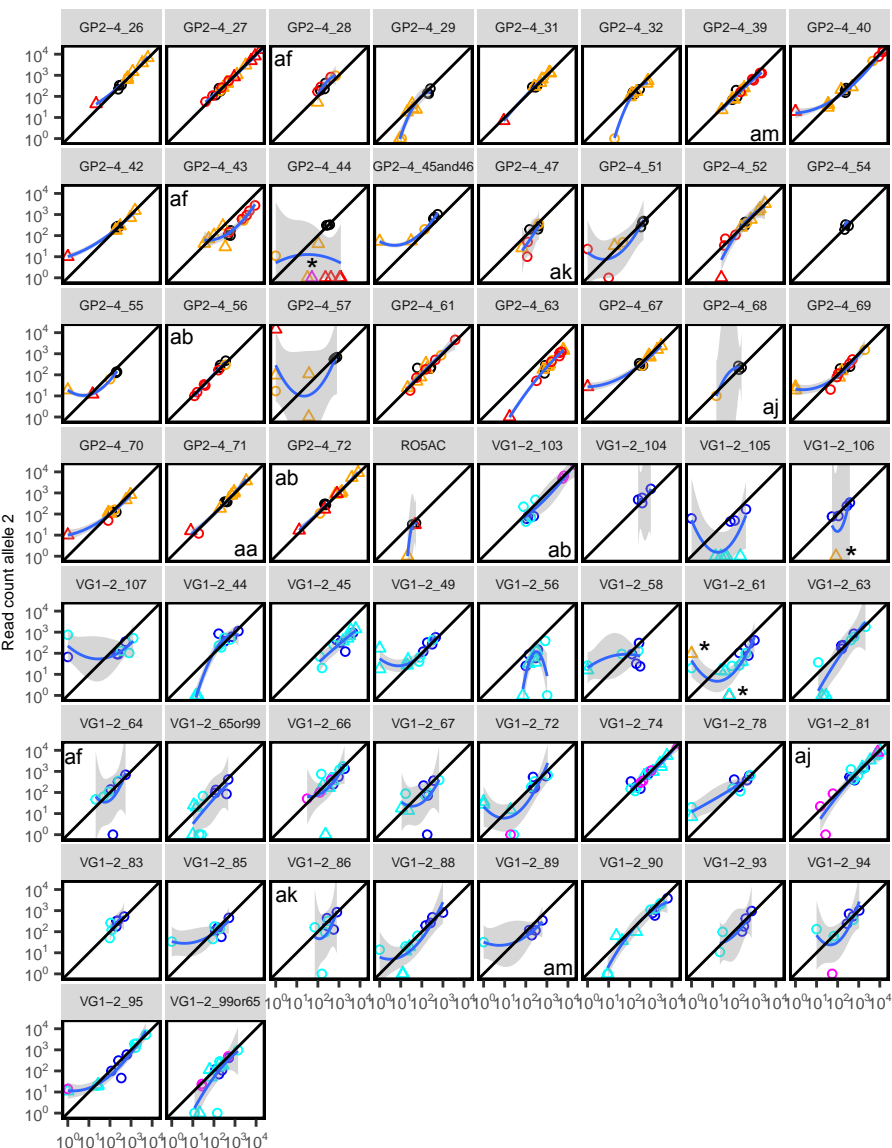

Read count allele 1

Supplement: Supplementary file 5 — Figure S3. [file MEN-25-e14067-s005.pdf]
