# Supplementary material for: Bamboozle: A Bioinformatic Tool for Identification and Quantification of Intraspecific Barcodes
Source: Mol Ecol Resour. 2025 Feb 4;25(4):e14067. doi: 10.1111/1755-0998.14067 (PMC11969633; doi:10.1111/1755-0998.14067)

Distribution of reads from sequencing
of C2W24 barcode in single-strain samples

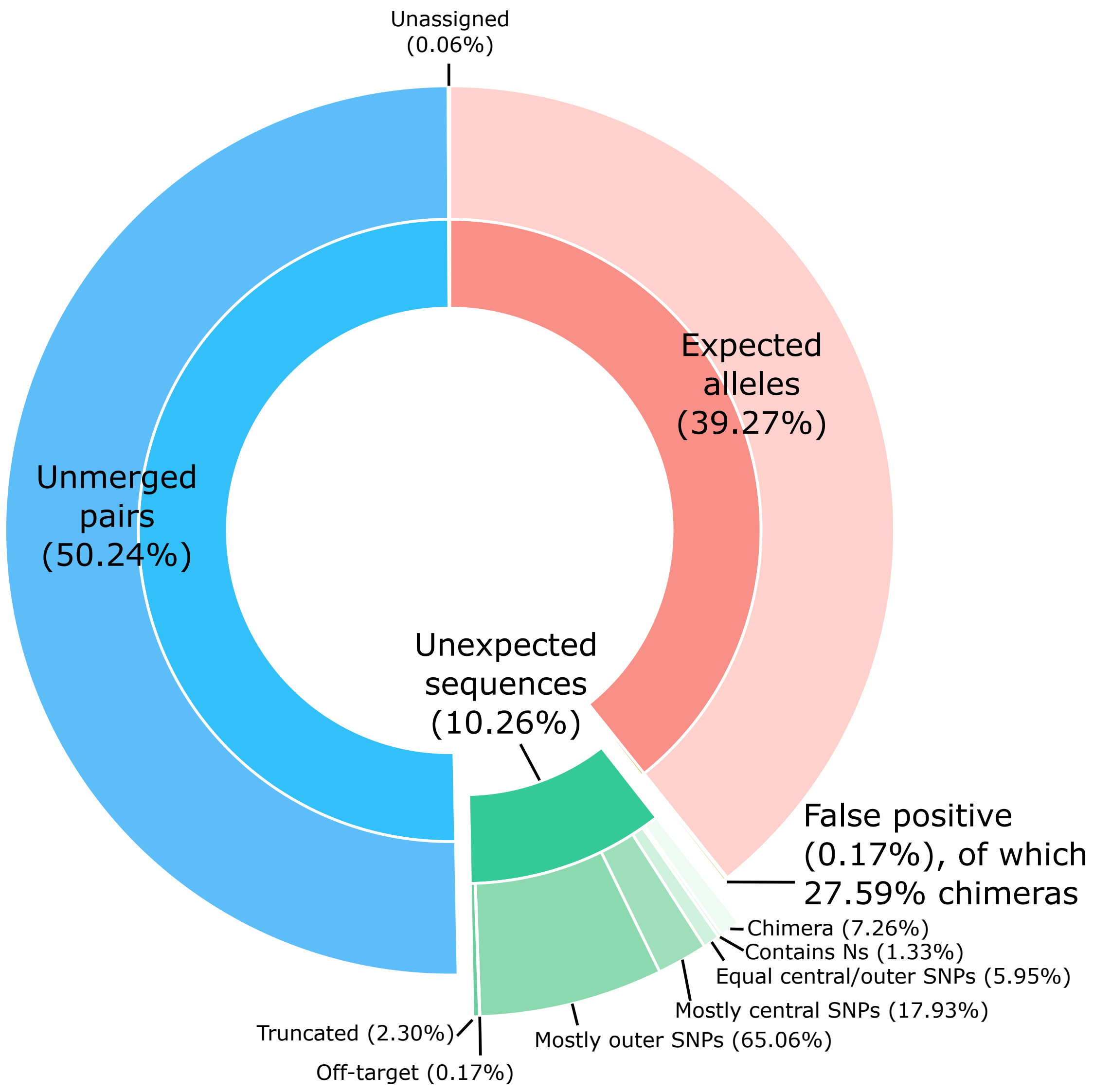

Distribution of reads from sequencing
of C12W1 barcode in single-strain samples

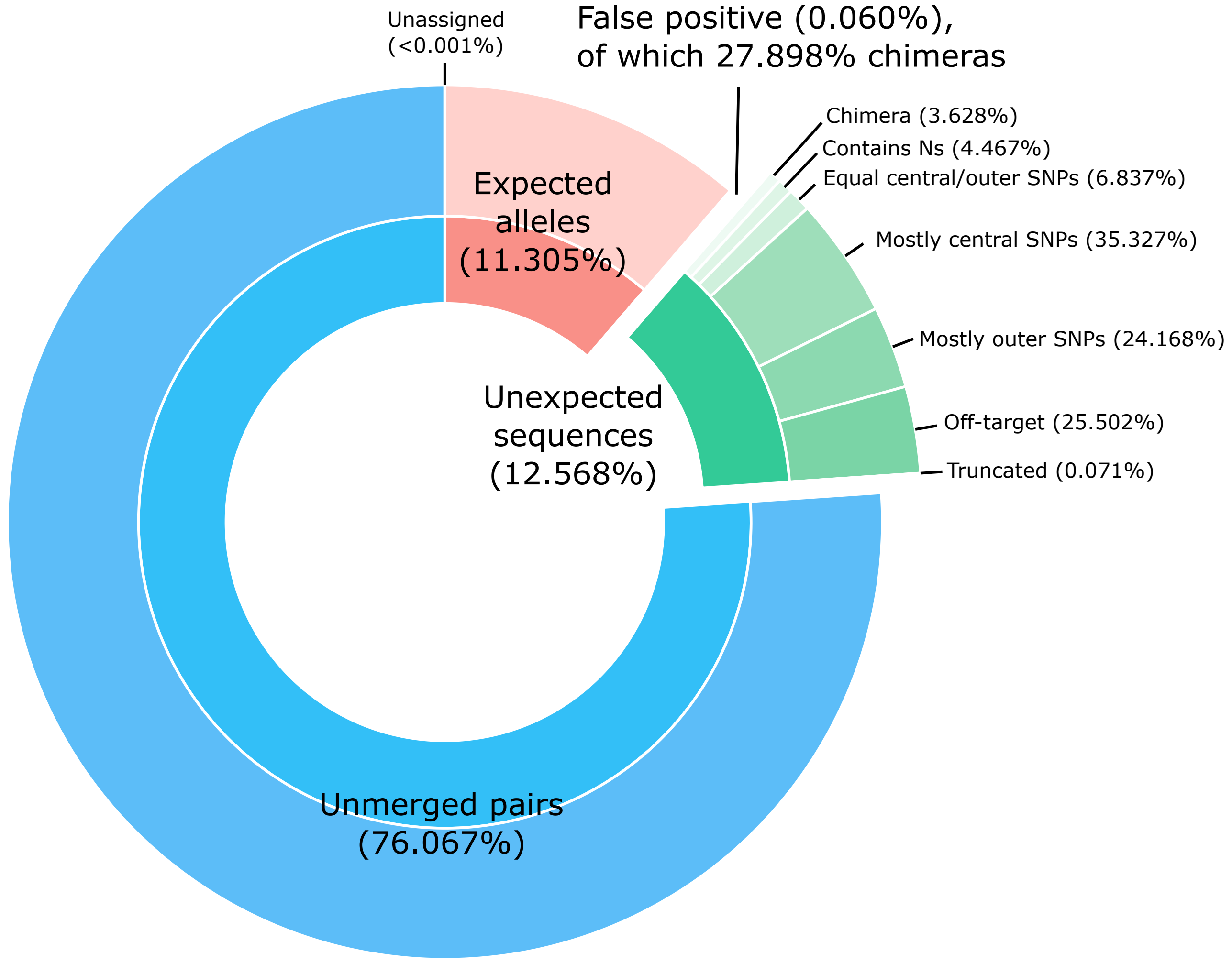

Distribution of reads from sequencing
of C12W2 barcode in single-strain samples

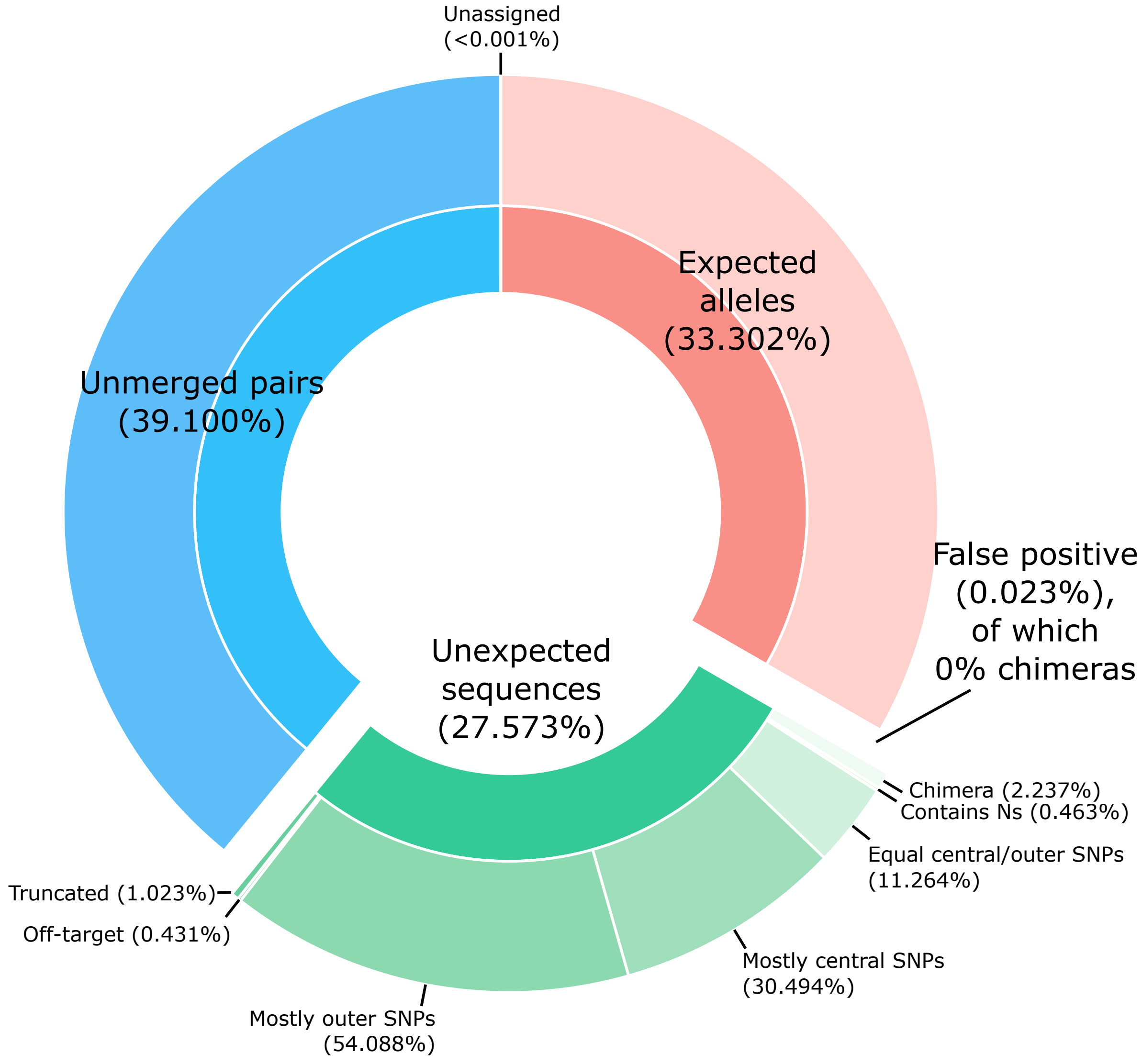

Distribution of reads from sequencing
of C16W4 barcode in single-strain samples

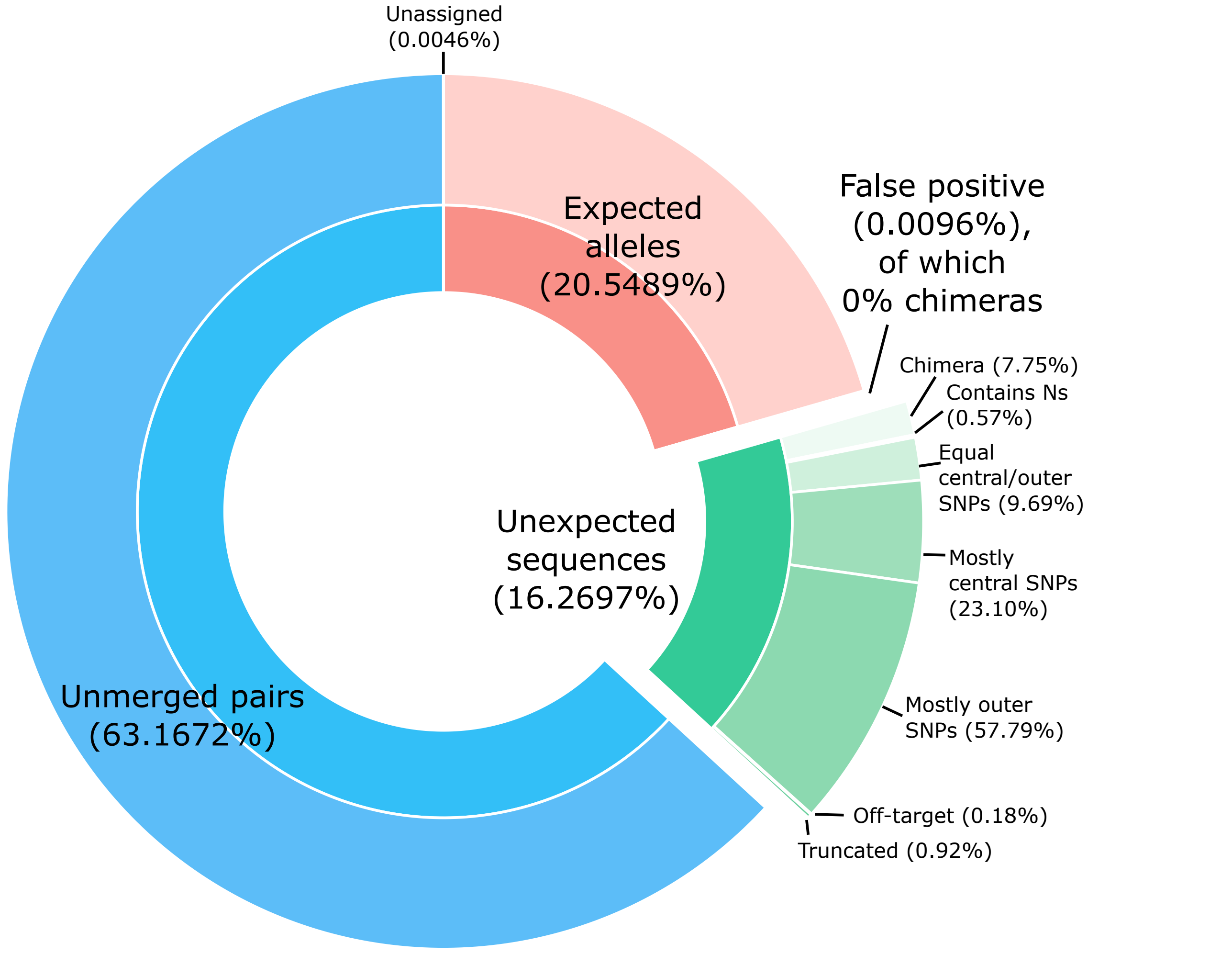

Supplement: Supplementary file 6 — Figure S4. [file MEN-25-e14067-s003.pdf]
